# Author Correction: Large language models are powerful electronic health record encoders

**DOI:** 10.1038/s41746-026-03220-1

**Published:** 2026-10-14

**Authors:** Stefan Hegselmann, Georg von Arnim, Tillmann Rheude, Noel Kronenberg, David Sontag, Gerhard Hindricks, Roland Eils, Benjamin Wild

**Affiliations:** 1 Berlin Institute of Health at Charité – Universitätsmedizin Berlin, Center of Digital Health, Berlin, Germany; 2 Berlin Institute of Health at Charité – Universitätsmedizin Berlin, BIH Biomedical Innovation Academy, BIH Charité Digital Clinician Scientist Program, Berlin, Germany; 3 Deutsches Herzzentrum der Charité (DHZC) – Universitätsmedizin Berlin, Berlin, Germany; 4 Freie Universität Berlin, Berlin, Germany; 5 Computer Science and Artificial Intelligence Laboratory (CSAIL), Massachusetts Institute of Technology (MIT), Cambridge, MA USA; 6 Layer Health Inc., Boston, MA USA; 7 Intelligent Medicine Institute, Fudan University, Shanghai, China; 8 Institute of Medical Informatics (IMI) – Universitätsmedizin Berlin, Berlin, Germany

**Keywords:** Prognosis, Computational models

Correction to: *npj Digital Medicine* https://doi.org/10.1038/s41746-026-02915-9, published online 06 July 2026

In the original version of this manuscript, there occur some errors consisting of numbers, typographical errors and formatting errors. The complete list of corrections is mentioned below. The original paper has now been corrected.

1. Outdated UK Biobank numbers in Figure 1 c) and Table 1. Table 1 should contain the following revised entries for the UK Biobank:
  UK Biobank No. of visits: 20,354,931 (instead of outdated 19,484,777)
  UK Biobank No. of events: 72,910,130 (instead of outdated 72,265,684)
  UK Biobank Black: 5715 (instead of wrong 5751)
2. Wrong model name in Figure 3, legend. The legend entry reads CLIMBR-T-Base; it should read CLMBR-T-Base.
3. Outdated UK Biobank number in Results, “Experimental setup”, p. 2.
  Published: “The processed UKB subset used in our study comprised 387,464 patients, approximately 19.5 million visits, [...]”
  Corrected: “The processed UKB subset used in our study comprised 387,464 patients, approximately 20.4 million visits, [...]”
4. Wrong table row referred to in Results, “LLM embeddings achieve strong performance in low-data regimes”, p. 4. The sentence quotes the UK Biobank win–loss counts in a paragraph about EHRSHOT.
  Published: “[...] outperformed CLMBR-T-Base on two versus zero tasks in the 8-shot setting and on four versus zero tasks in the 64-shot setting, while most tasks remained indistinguishable (Table 4).”
  Corrected: “[...] outperformed CLMBR-T-Base on four versus one tasks in the 8-shot setting and on three versus two tasks in the 64-shot setting, while most tasks remained indistinguishable (Table 4).”
5. Former result referred to in Results, “General-purpose LLM embeddings rival domain-specific EHR models”, p. 4. The sentence refers to the old BioClinicalBERT MEME results instead of the updated BioClinicalBERT.
  Published: “[...] BioClinicalBERT with mean embeddings over 512-token chunks performed best at 0.702 (0.671–0.732) (Tables 3 and S4).”
  Corrected: “[...] BioClinicalBERT with mean embeddings over 512-token chunks performed best at 0.705 (0.680–0.730) (Tables 3 and S4).”
6. Transposed values in Supplementary Table S6. The chest X-ray values for the two “Counts + TB” rows are transposed between the GBM and LR blocks.
7. Imprecise wording in Discussion, limitations, p. 9. Specified hyperparameter tuning and replaced wrong usage of “two-shot settings” where “two shot sizes” was meant.
  Published: “In addition, our fine-tuning experiments were limited to two task groups and two-shot settings due [...]”
  Corrected: “In addition, the hyperparameter tuning for our fine-tuning experiments was limited to two task groups and two shot sizes due [...]”
8. Wrong number of attention heads stated for DeBERTa v3 large model in Methods, “LLM embedding models and baselines”, p. 11.
  Published: “[...] large variant (24 layers, 12 attention heads, 1024 hidden size), with [...]”
  Corrected: “[...] large variant (24 layers, 16 attention heads, 1024 hidden size), with [...]”
9. Wrong use of “an encoder” instead of “a decoder” in Methods, “Existing methods using language models for EHR prediction”, p. 12.
  Published: “They also evaluate an encoder-based approach producing Yes and No tokens [...]”
  Corrected: “They also evaluate a decoder-based approach producing Yes and No tokens [...]”
10. Outdated UK Biobank numbers in Methods, “External validation on UK Biobank” p.13.
  Published: “[...] increasing the number of unique codes from 51,677 to 69,850, and [...]”
  Corrected: “[...] increasing the number of unique codes from 50,702 to 71,393, and [...]”
11. Wrong task name in Methods, “Statistical testing”, p. 13. Used task name “CheXpert” while task was introduced as “Chest X-ray findings”.
  Published: “For the CheXpert task, which [...]”
  Corrected: “For the chest X-ray findings task, which [...]”
12. Missing space in the “Author contributions”, p. 16.

Published: “[...] and B.W.S.H. drafted the initial manuscript, [...]”

Corrected: “[...] and B.W. S.H. drafted the initial manuscript, [...]”

