# Supplementary material for: Large language models are powerful electronic health record encoders
Source: NPJ Digit Med. 2026 Jul 6;9:530. doi: 10.1038/s41746-026-02915-9 (PMC13356027; doi:10.1038/s41746-026-02915-9)
Supplement: Supplementary file 1 — Supplementary Information [file 41746_2026_2915_MOESM1_ESM.pdf]

1 Supplementary Information

1.1 Additional Experimental Details

Table S1 UK Biobank Prediction Tasks Overview. The external validation on the UKB includes three task groups. Operational outcomes and mortality prediction only consist of a single prediction task, while the assignment of new diagnosis spans 23 conditions. Following [45], canonical splits for training, validation, and testing were defined.

**Attribute**	**Train Labels (Positive)**	**Valid Labels (Positive)**	**Test Labels (Positive)**	**Total Labels (Positive)**
**Operational Outcomes**				
Hospitalization	119463 (22992)	119463 (22913)	119463 (22624)	358389 (68529)
**Mortality Prediction**				
Death	119459 (268)	119463 (284)	119462 (250)	358384 (802)
**Assignment of New Diagnoses**				
Hypertension	100049 (2515)	99866 (2532)	99613 (2600)	299528 (7647)
Diabetes Mellitus	114423 (621)	114531 (616)	114380 (604)	343334 (1841)
Atrial Fibrillation	118804 (107)	118784 (94)	118704 (105)	356292 (306)
Pneumonia	117409 (270)	117527 (299)	117437 (232)	352373 (801)
Chronic Obstructive Pulmonary Disease [COPD]	117868 (305)	117899 (284)	117886 (334)	353653 (923)
Chronic Kidney Disease	117542 (308)	117512 (346)	117478 (347)	352532 (1001)
Ischemic Heart Disease	113627 (609)	113634 (583)	113467 (596)	340728 (1788)
Myocardial Infarction [heart Attack]	117049 (214)	117070 (204)	116995 (201)	351114 (619)
Cerebral Infarction [ischemic Stroke]	118788 (100)	118788 (116)	118745 (94)	356321 (310)
Heart Failure	118563 (152)	118626 (158)	118609 (125)	355798 (435)
Cardiac Arrest	119370 (25)	119362 (29)	119370 (33)	358102 (87)
Abdominal Aortic Aneurysm	119359 (17)	119352 (17)	119354 (39)	358065 (73)
Pulmonary Embolism	118750 (81)	118795 (99)	118754 (68)	356299 (248)
Aortic Stenosis	119170 (38)	119174 (37)	119179 (39)	357523 (114)
Mitral Valve Insufficiency	119003 (63)	119061 (58)	119024 (65)	357088 (186)
Endocarditis	119005 (24)	119012 (24)	118998 (31)	357015 (79)
Rheumatic Fever And Chronic Rheumatic Heart Diseases	119047 (51)	119073 (46)	119083 (57)	357203 (154)
Anemia	114135 (606)	114119 (587)	114142 (620)	342396 (1813)
Back Pain	100129 (1323)	99663 (1322)	99876 (1285)	299668 (3930)
Parkinson's Disease (primary)	119278 (27)	119306 (31)	119284 (28)	357868 (86)
Rheumatoid Arthritis	118392 (117)	118400 (107)	118380 (92)	355172 (316)
Psoriasis	117482 (148)	117461 (119)	117594 (142)	352537 (409)
Suicide Ideation And Attempt Or Self Harm	118953 (62)	118896 (60)	118943 (53)	356792 (175)

Table S2 Semantic Codes for Aggregated Concepts in EHR Markdown Serialization. We aggregated time-series data encoded via LOINC concepts and identified the most frequent concepts from which we selected 24 key medical concepts. To reduce duplicate information, we merged synonymous semantic codes. The primary LOINC code is presented first in the column Semantic Codes followed by identified duplicates. We also defined a unit, minimum and maximum allowed values for filtering, a normal range to classify values in low, normal, and high, and a formatting strategy to create our EHR serialization.

Medical Concept	Semantic Codes	Unit	Min-Max Range	Normal Range	Formatting
**Recent Body Metrics**					
Body weight	LOINC/29463-7	oz	350-10000		One decimal
Body height	LOINC/8302-2	inch	5-100		One decimal
Body mass index / BMI	LOINC/39156-5	kg/m2	10-100	18.5-24.9	One decimal
Body surface area	LOINC/8277-6, SNOMED/301898006	m2	0.1-10		Two decimals
**Recent Vital Signs**					
Heart rate	LOINC/8867-4, SNOMED/364075005, SNOMED/78564009	bpm	5-300	60-100	Integer
Systolic blood pressure	LOINC/8480-6, SNOMED/271649006	mmHg	20-300	90-140	Integer
Diastolic blood pressure	LOINC/8462-4, SNOMED/271650006	mmHg	20-300	60-90	Integer
Body temperature	LOINC/8310-5	°F	80-120	95-100.4	One decimal
Respiratory rate	LOINC/9279-1	breaths/min	1-100	12-18	Integer
Oxygen saturation	LOINC/LP21258-6	%	1-100	95-100	Integer
**Recent Lab Results**					
Hemoglobin	LOINC/718-7, SNOMED/271026005, SNOMED/441689006	g/dL	1-20	12-17	One decimal
Hematocrit	LOINC/4544-3, LOINC/20570-8, LOINC/48703-3, SNOMED/28317006	%	10-100	36-51	Integer
Erythrocytes	LOINC/789-8, LOINC/26453-1	106/uL	1-10	4.2-5.9	Two decimals
Leukocytes	LOINC/20584-9, LOINC/6690-2	103/uL	1-100	4-10	One decimal
Platelets	LOINC/777-3, SNOMED/61928009	103/uL	10-1000	150-350	Integer
Sodium	LOINC/2951-2, LOINC/2947-0, SNOMED/25197003	mmol/L	100-200	136-145	Integer
Potassium	LOINC/2823-3, SNOMED/312468003, LOINC/6298-4, SNOMED/59573005	mmol/L	0.1-10	3.5-5.0	One decimal
Chloride	LOINC/2075-0, SNOMED/104589004, LOINC/2069-3	mmol/L	50-200	98-106	Integer
Carbon dioxide, total	LOINC/2028-9	mmol/L	10-100	23-28	Integer
Calcium	LOINC/17861-6, SNOMED/271240001	mg/dL	1-20	9-10.5	One decimal
Glucose	LOINC/2345-7, SNOMED/166900001, LOINC/2339-0, SNOMED/33747003, LOINC/14749-6	mg/dL	10-1000	70-100	Integer
Urea nitrogen	LOINC/3094-0, SNOMED/105011006	mg/dL	1-200	8-20	Integer
Creatinine	LOINC/2160-0, SNOMED/113075003	mg/dL	0.1-10	0.7-1.3	One decimal
Anion gap	LOINC/33037-3, LOINC/41276-7, SNOMED/25469001	mmol/L	-20-50	3-11	Integer

Table S3 Instructions for LLM Embedding Models. The LLM embedding models were trained using instructions; hence, we also defined simple task-specific prompts for each of the 15 clinical prediction tasks. Each prompt is prepended by the prefix given below, containing a general task description. The three tasks used for the external validation on UKB use a similar design. For the decoder model we added an additional instruction to the prompt enforcing the output of `Yes` and `No` tokens used for prediction.

**Task**	**Prompt**
Prefix (for all tasks)	Given a patient's electronic healthcare record (EHR) as a newline separated list, retrieve relevant passages that answer the query:
**EHRSHOT**	
Long Length of Stay	will the patient stay in the hospital for more than 7 days
30-day Readmission	will the patient be readmitted to the hospital within 30 days
ICU Transfer	will the patient be transferred to the intensive care unit
Thrombocytopenia	has the patient thrombocytopenia
Hyperkalemia	has the patient hyperkalemia
Hypoglycemia	has the patient hypoglycemia
Hyponatremia	has the patient hyponatremia
Anemia	has the patient anemia
Hypertension	has the patient hypertension
Hyperlipidemia	has the patient hyperlipidemia
Pancreatic Cancer	has the patient pancreatic cancer
Celiac	has the patient celiac disease
Lupus	has the patient lupus
Acute MI	has the patient an acute myocardial infarction
Chest X-Ray Findings	what are the chest x-ray findings of the patient
Generic (ablation)	what are the key clinical features of the patient to predict future medical events
**UK Biobank (UKB)**	
Mortality Prediction	will the patient die within one year
Hospitalization	will the patient be admitted to the hospital within one year
Assignment of New Diagnoses	has the patient {diagnosis name}
**Additional Prompt Added for Decoder Qwen3-8B**	
Decoder Prompt	Answer STRICTLY with a single token: Yes or No. No punctuation, no extra words.

1.2 Full Results on EHRSHOT

Table S4 Performance for All Examples on EHRSHOT. Mean area under the receiver operating characteristic curve (AUROC) performance with approximate 95% confidence intervals of all included models for four task groups. The macro-averaged performance across all task groups is given in the right-most column. All LLM embedding models use a context size of 8,192 tokens.

Model	Operational Outcomes	Anticipating Lab Test Results	Assignment of New Diagnosis	Anticipating Chest X-ray Findings	Macro Avg. Across Task Groups
**Baselines** [45]					
CLMBR-T-Base	$0.824_{.803-.845}$	$0.832_{.824-.840}$	$0.707_{.667-.746}$	$0.713_{.702-.724}$	$0.769_{.746-.792}$
Count-based + GBM	$0.824_{.804-.844}$	$0.841_{.833-.849}$	$0.758_{.724-.793}$	$0.686_{.674-.699}$	$0.777_{.756-.799}$
Count-based + LR	$0.764_{.741-.787}$	$0.742_{.729-.756}$	$0.734_{.687-.782}$	$0.673_{.656-.690}$	$0.728_{.700-.757}$
**LLM Embedding Models**					
Qwen3-Emb-8B	$0.797_{.773-.820}$	$0.842_{.835-.850}$	$0.714_{.672-.757}$	$0.722_{.711-.733}$	$0.769_{.744-.794}$
Qwen3-Emb-4B	$0.787_{.764-.810}$	$0.824_{.816-.831}$	$0.718_{.667-.768}$	$0.708_{.696-.719}$	$0.759_{.730-.787}$
Qwen3-Emb-0.6B	$0.778_{.753-.803}$	$0.742_{.732-.753}$	$0.684_{.631-.737}$	$0.705_{.694-.716}$	$0.727_{.697-.758}$
Qwen2-Emb-7B	$0.772_{.749-.796}$	$0.746_{.735-.757}$	$0.744_{.705-.784}$	$0.685_{.671-.699}$	$0.737_{.712-.761}$
Qwen2-Emb-1.5B	$0.756_{.735-.778}$	$0.710_{.699-.721}$	$0.696_{.647-.744}$	$0.680_{.668-.691}$	$0.710_{.683-.738}$
Llama3.1-LLM2Vec-8B	$0.763_{.738-.787}$	$0.726_{.714-.738}$	$0.727_{.688-.766}$	$0.686_{.673-.699}$	$0.725_{.701-.750}$
**LLM Embedding Model + EHR Foundation Model** [45]					
Qwen3-Emb-8B + CLMBR-T-Base	$0.821_{.800-.842}$	$0.864_{.858-.871}$	$0.736_{.695-.777}$	$0.731_{.721-.742}$	$0.788_{.764-.812}$
**LLM Embedding Model + GBM Head**					
Qwen3-Emb-8B + GBM	$0.774_{.749-.799}$	$0.812_{.804-.820}$	$0.685_{.644-.727}$	$0.696_{.686-.706}$	$0.742_{.717-.767}$
**Multiple Embedding Model for EHR (MEME)** [34] **with Linear Head**					
Qwen3-Emb-8B MEME	$0.814_{.793-.834}$	$0.845_{.837-.852}$	$0.728_{.673-.784}$	$0.717_{.705-.728}$	$0.776_{.746-.806}$
BioClinicalBERT MEME	$0.756_{.733-.778}$	$0.699_{.686-.713}$	$0.704_{.651-.758}$	$0.648_{.635-.661}$	$0.702_{.671-.732}$
MedBERT MEME	$0.759_{.736-.782}$	$0.695_{.682-.708}$	$0.715_{.676-.755}$	$0.620_{.605-.635}$	$0.697_{.673-.722}$
**Encoder Language Models with Mean Embeddings of Chunked Inputs**					
BioClinicalBERT	$0.738_{.712-.763}$	$0.698_{.685-.711}$	$0.707_{.668-.746}$	$0.679_{.666-.691}$	$0.705_{.680-.730}$
MedBERT	$0.742_{.718-.767}$	$0.694_{.683-.706}$	$0.663_{.614-.713}$	$0.683_{.671-.696}$	$0.696_{.667-.725}$
DeBERTaV3 large	$0.737_{.713-.762}$	$0.694_{.681-.706}$	$0.676_{.629-.724}$	$0.660_{.648-.673}$	$0.692_{.664-.720}$
DeBERTaV3 base	$0.727_{.701-.753}$	$0.680_{.668-.693}$	$0.672_{.635-.710}$	$0.659_{.647-.672}$	$0.685_{.660-.709}$
BERT large	$0.748_{.725-.771}$	$0.688_{.675-.700}$	$0.671_{.625-.718}$	$0.669_{.656-.682}$	$0.694_{.667-.722}$
BERT base	$0.748_{.724-.772}$	$0.693_{.681-.706}$	$0.685_{.641-.729}$	$0.677_{.664-.690}$	$0.701_{.674-.727}$
**Encoder Language Models with Concatenated Embeddings of Chunked Inputs**					
BioClinicalBERT	$0.740_{.715-.764}$	$0.707_{.696-.718}$	$0.690_{.651-.730}$	$0.653_{.641-.665}$	$0.698_{.673-.722}$
MedBERT	$0.751_{.726-.775}$	$0.712_{.701-.723}$	$0.658_{.618-.698}$	$0.658_{.646-.670}$	$0.695_{.670-.720}$
DeBERTaV3 large	$0.714_{.687-.742}$	$0.690_{.679-.701}$	$0.632_{.595-.669}$	$0.624_{.612-.636}$	$0.665_{.641-.690}$
DeBERTaV3 base	$0.675_{.647-.703}$	$0.680_{.670-.690}$	$0.623_{.583-.662}$	$0.615_{.603-.628}$	$0.648_{.623-.674}$
BERT large	$0.736_{.710-.762}$	$0.697_{.686-.708}$	$0.657_{.613-.701}$	$0.643_{.631-.655}$	$0.683_{.657-.710}$
BERT base	$0.752_{.728-.776}$	$0.705_{.695-.716}$	$0.657_{.613-.701}$	$0.650_{.639-.662}$	$0.691_{.665-.717}$

Table S5 Per-task ΔAUROC (Qwen3-Emb-8B minus baseline) on EHRSHOT. Cells report the AUROC difference (ΔAUROC), 95% bootstrap confidence intervals, and Holm-adjusted p-values obtained from paired patient-level bootstrap tests with 10,000 resamples. Positive values indicate better performance of Qwen3-Emb-8B. Multiple testing was controlled separately for each shot setting ($k = 8$, $k = 64$, and all training data) using Holm's procedure across all tasks and baseline comparisons (45 tests per setting). Bold indicates statistically significant differences ($p_{\text{adj}} < 0.05$).

Task	CLMBR-T-Base	BioClinicalBERT	Count-based Model
**8-shot (8 positive / 8 negative examples)**			
Long LOS	+0.011 [−0.025,+0.047] (1.000)	−0.001 [−0.034,+0.031] (1.000)	−0.042 [−0.080,−0.002] (0.677)
30-day Readmission	+0.001 [−0.034,+0.034] (1.000)	**+0.079** [+0.038,+0.121] (0.009)	**+0.085** [+0.039,+0.131] (0.009)
ICU Transfer	−0.043 [−0.089,+0.002] (1.000)	+0.071 [+0.009,+0.132] (0.572)	+0.069 [−0.015,+0.151] (1.000)
Thrombocytopenia	**+0.036** [+0.014,+0.057] (0.041)	**+0.089** [+0.068,+0.110] (0.009)	**+0.100** [+0.074,+0.123] (0.009)
Hyperkalemia	**+0.113** [+0.082,+0.141] (0.009)	**+0.197** [+0.160,+0.236] (0.009)	**+0.194** [+0.146,+0.241] (0.009)
Hypoglycemia	−0.042 [−0.080,−0.004] (0.584)	+0.050 [+0.009,+0.089] (0.415)	+0.024 [−0.021,+0.067] (1.000)
Hyponatremia	**+0.029** [+0.011,+0.047] (0.041)	**+0.085** [+0.061,+0.109] (0.009)	**+0.070** [+0.046,+0.095] (0.009)
Anemia	**−0.067** [−0.080,−0.056] (0.009)	**+0.145** [+0.130,+0.161] (0.009)	**+0.032** [+0.021,+0.043] (0.009)
Hypertension	−0.068 [−0.125,−0.012] (0.415)	−0.012 [−0.060,+0.037] (1.000)	+0.002 [−0.065,+0.070] (1.000)
Hyperlipidemia	+0.019 [−0.028,+0.069] (1.000)	+0.030 [−0.027,+0.088] (1.000)	+0.076 [+0.011,+0.141] (0.474)
Pancreatic Cancer	+0.089 [+0.008,+0.174] (0.600)	+0.071 [−0.024,+0.164] (1.000)	**+0.167** [+0.074,+0.259] (0.030)
Celiac	+0.070 [−0.113,+0.299] (1.000)	+0.209 [−0.166,+0.453] (1.000)	+0.009 [−0.262,+0.232] (1.000)
Lupus	+0.054 [−0.088,+0.184] (1.000)	+0.123 [−0.005,+0.270] (1.000)	+0.063 [−0.097,+0.237] (1.000)
Acute MI	**+0.126** [+0.056,+0.191] (0.025)	**+0.161** [+0.081,+0.237] (0.009)	**+0.172** [+0.088,+0.251] (0.009)
Chest X-ray Findings	−0.015 [−0.028,−0.001] (0.600)	+0.024 [+0.009,+0.037] (0.062)	**+0.026** [+0.010,+0.042] (0.043)
**64-shot (64 positive / 64 negative examples)**			
Long LOS	−0.037 [−0.060,−0.014] (0.054)	+0.019 [−0.011,+0.049] (1.000)	−0.025 [−0.054,+0.004] (1.000)
30-day Readmission	−0.010 [−0.029,+0.009] (1.000)	**+0.050** [+0.030,+0.069] (0.009)	+0.022 [+0.002,+0.041] (0.846)
ICU Transfer	−0.069 [−0.123,−0.014] (0.418)	+0.056 [−0.007,+0.120] (1.000)	−0.041 [−0.097,+0.016] (1.000)
Thrombocytopenia	**+0.023** [+0.009,+0.036] (0.026)	**+0.078** [+0.066,+0.092] (0.009)	**−0.047** [−0.060,−0.036] (0.009)
Hyperkalemia	**+0.044** [+0.026,+0.062] (0.009)	**+0.143** [+0.115,+0.169] (0.009)	+0.020 [−0.001,+0.040] (1.000)
Hypoglycemia	**−0.044** [−0.070,−0.018] (0.043)	**+0.094** [+0.057,+0.131] (0.009)	+0.034 [+0.002,+0.065] (1.000)
Hyponatremia	+0.016 [−0.003,+0.035] (1.000)	**+0.118** [+0.097,+0.137] (0.009)	**−0.072** [−0.091,−0.055] (0.009)
Anemia	**−0.056** [−0.064,−0.049] (0.009)	**+0.156** [+0.141,+0.172] (0.009)	+0.004 [−0.003,+0.011] (1.000)
Hypertension	−0.038 [−0.073,−0.002] (1.000)	+0.028 [−0.018,+0.074] (1.000)	+0.002 [−0.040,+0.045] (1.000)
Hyperlipidemia	+0.030 [−0.009,+0.068] (1.000)	+0.037 [−0.013,+0.087] (1.000)	+0.020 [−0.021,+0.063] (1.000)
Pancreatic Cancer	**+0.090** [+0.038,+0.148] (0.038)	+0.073 [+0.001,+0.141] (1.000)	+0.011 [−0.042,+0.071] (1.000)
Celiac	+0.101 [−0.080,+0.257] (1.000)	+0.169 [−0.121,+0.317] (1.000)	−0.029 [−0.138,+0.100] (1.000)
Lupus	−0.079 [−0.205,+0.040] (1.000)	+0.019 [−0.122,+0.163] (1.000)	+0.039 [−0.133,+0.220] (1.000)
Acute MI	−0.028 [−0.066,+0.010] (1.000)	−0.047 [−0.089,−0.002] (1.000)	−0.041 [−0.083,+0.002] (1.000)
Chest X-ray Findings	+0.000 [−0.009,+0.010] (1.000)	**+0.031** [+0.018,+0.045] (0.009)	**+0.021** [+0.010,+0.032] (0.014)
**All training data**			
Long LOS	−0.006 [−0.024,+0.012] (1.000)	**+0.079** [+0.058,+0.100] (0.009)	−0.018 [−0.035,−0.001] (0.840)
30-day Readmission	−0.015 [−0.037,+0.007] (1.000)	**+0.045** [+0.025,+0.067] (0.011)	−0.002 [−0.029,+0.026] (1.000)
ICU Transfer	−0.060 [−0.110,−0.012] (0.463)	+0.053 [+0.005,+0.103] (0.638)	−0.062 [−0.115,−0.007] (0.658)
Thrombocytopenia	**+0.045** [+0.035,+0.054] (0.009)	**+0.116** [+0.100,+0.132] (0.009)	**−0.040** [−0.049,−0.032] (0.009)
Hyperkalemia	**+0.030** [+0.015,+0.045] (0.009)	**+0.142** [+0.114,+0.172] (0.009)	+0.022 [+0.006,+0.038] (0.178)
Hypoglycemia	**−0.035** [−0.053,−0.019] (0.009)	**+0.146** [+0.115,+0.176] (0.009)	**+0.061** [+0.036,+0.086] (0.009)
Hyponatremia	**+0.034** [+0.022,+0.046] (0.009)	**+0.179** [+0.163,+0.195] (0.009)	**−0.027** [−0.037,−0.017] (0.009)
Anemia	**−0.021** [−0.023,−0.018] (0.009)	**+0.139** [+0.126,+0.153] (0.009)	**−0.009** [−0.012,−0.006] (0.009)
Hypertension	−0.042 [−0.084,−0.002] (0.840)	+0.036 [−0.009,+0.082] (1.000)	−0.033 [−0.079,+0.011] (1.000)
Hyperlipidemia	+0.022 [−0.017,+0.063] (1.000)	+0.017 [−0.024,+0.057] (1.000)	−0.018 [−0.064,+0.028] (1.000)
Pancreatic Cancer	+0.049 [+0.002,+0.100] (0.840)	+0.052 [−0.012,+0.110] (1.000)	−0.022 [−0.072,+0.019] (1.000)
Celiac	+0.023 [−0.153,+0.171] (1.000)	−0.118 [−0.281,−0.019] (0.565)	−0.130 [−0.322,+0.009] (1.000)
Lupus	−0.019 [−0.134,+0.088] (1.000)	+0.059 [−0.046,+0.174] (1.000)	−0.070 [−0.206,+0.059] (1.000)
Acute MI	+0.014 [−0.015,+0.042] (1.000)	−0.003 [−0.037,+0.029] (1.000)	+0.012 [−0.029,+0.053] (1.000)
Chest X-ray Findings	+0.009 [−0.001,+0.018] (1.000)	**+0.043** [+0.031,+0.057] (0.009)	**+0.036** [+0.023,+0.048] (0.009)

Table S6 Performance for All Examples on EHRSHOT Across Different Count-based Models. Mean area under the receiver operating characteristic curve (AUROC) performance with approximate 95% confidence intervals for the count-based model from [45] using ontology expansion. We tested a GBM and LR model and extensions including the encoding of string values (SV), numeric values (NV), time binning with four time bins (TB), and the extension with all three.

**Model**	**Operational Outcomes**	**Anticipating Lab Test Results**	**Assignment of New Diagnosis**	**Anticipating Chest X-ray Findings**	**Macro Avg. Across Task Groups**
**GBM Model**					
Counts [45]	0.774 $_{.752-.797}$	0.728 $_{.716-.741}$	0.719 $_{.669-.768}$	0.656 $_{.641-.671}$	0.719 $_{.691-.748}$
Counts + SV	0.785 $_{.764-.806}$	0.731 $_{.719-.744}$	0.732 $_{.685-.779}$	0.662 $_{.649-.675}$	0.727 $_{.700-.755}$
Counts + NV	0.786 $_{.763-.809}$	0.789 $_{.779-.800}$	0.752 $_{.711-.792}$	0.656 $_{.642-.670}$	0.746 $_{.721-.771}$
Counts + TB	0.815 $_{.795-.836}$	0.752 $_{.741-.764}$	0.768 $_{.736-.801}$	0.666 $_{.650-.683}$	0.756 $_{.735-.776}$
Counts + SV/NV/TB	0.824 $_{.804-.844}$	0.841 $_{.833-.849}$	0.758 $_{.724-.793}$	0.686 $_{.674-.699}$	0.777 $_{.756-.799}$
**LR Model**					
Counts [45]	0.719 $_{.692-.746}$	0.669 $_{.653-.685}$	0.749 $_{.711-.787}$	0.646 $_{.633-.660}$	0.696 $_{.670-.721}$
Counts + SV	0.720 $_{.693-.747}$	0.669 $_{.654-.685}$	0.750 $_{.712-.788}$	0.647 $_{.633-.661}$	0.696 $_{.671-.722}$
Counts + NV	0.718 $_{.691-.746}$	0.687 $_{.671-.703}$	0.752 $_{.715-.789}$	0.655 $_{.641-.669}$	0.703 $_{.678-.728}$
Counts + TB	0.763 $_{.741-.785}$	0.717 $_{.703-.730}$	0.741 $_{.695-.787}$	0.686 $_{.674-.698}$	0.722 $_{.694-.749}$
Counts + SV/NV/TB	0.764 $_{.741-.787}$	0.742 $_{.729-.756}$	0.734 $_{.687-.782}$	0.673 $_{.656-.690}$	0.728 $_{.700-.757}$

Table S7 Performance for All Examples on EHRSHOT for Different Serializations. Mean area under the receiver operating characteristic curve (AUROC) performance with approximate 95% confidence intervals of the list serialization used in this work and three alternatives using the first occurrences of each code and adding timestamps to each code. We also tested a handcrafted Markdown EHR serialization with three LLMs and JSON, XML, and YAML data formats with Qwen3-Emb-8B.

Model	Operational Outcomes	Anticipating Lab Test Results	Assignment of New Diagnosis	Anticipating Chest X-ray Findings	Macro Avg. Across Task Groups
**EHR List Serializations for Qwen3-Emb-8B**					
List codes recent (ours)	0.797 $_{.773\text{-}.820}$	0.842 $_{.835\text{-}.850}$	0.714 $_{.672\text{-}.757}$	0.722 $_{.711\text{-}.733}$	0.769 $_{.744\text{-}.794}$
List codes first	0.761 $_{.736\text{-}.785}$	0.715 $_{.701\text{-}.728}$	0.731 $_{.688\text{-}.773}$	0.676 $_{.663\text{-}.690}$	0.721 $_{.694\text{-}.747}$
List codes recent + time	0.795 $_{.772\text{-}.818}$	0.844 $_{.837\text{-}.851}$	0.692 $_{.637\text{-}.748}$	0.727 $_{.716\text{-}.738}$	0.765 $_{.734\text{-}.795}$
List codes first + time	0.746 $_{.720\text{-}.772}$	0.703 $_{.689\text{-}.716}$	0.718 $_{.674\text{-}.761}$	0.645 $_{.629\text{-}.660}$	0.703 $_{.675\text{-}.730}$
**EHR Markdown Serializations**					
Qwen3-Emb-8B	0.773 $_{.749\text{-}.797}$	0.859 $_{.852\text{-}.866}$	0.725 $_{.683\text{-}.767}$	0.694 $_{.681\text{-}.707}$	0.763 $_{.737\text{-}.788}$
Qwen2-Emb-7B	0.756 $_{.731\text{-}.781}$	0.767 $_{.756\text{-}.778}$	0.717 $_{.671\text{-}.764}$	0.677 $_{.664\text{-}.690}$	0.729 $_{.702\text{-}.757}$
Llama3.1-LLM2Vec-8B	0.769 $_{.746\text{-}.792}$	0.732 $_{.720\text{-}.744}$	0.705 $_{.651\text{-}.759}$	0.692 $_{.679\text{-}.705}$	0.724 $_{.694\text{-}.755}$
**EHR Alternative Serialization Formats for Qwen3-Emb-8B**					
JSON	0.773 $_{.749\text{-}.796}$	0.858 $_{.851\text{-}.865}$	0.736 $_{.692\text{-}.780}$	0.690 $_{.677\text{-}.704}$	0.764 $_{.738\text{-}.790}$
XML	0.771 $_{.747\text{-}.795}$	0.862 $_{.855\text{-}.868}$	0.726 $_{.681\text{-}.771}$	0.676 $_{.663\text{-}.690}$	0.759 $_{.732\text{-}.785}$
YAML	0.773 $_{.749\text{-}.796}$	0.863 $_{.856\text{-}.870}$	0.723 $_{.677\text{-}.769}$	0.684 $_{.670\text{-}.698}$	0.761 $_{.734\text{-}.787}$

Table S8 Code Categories Used for Ablation Study. For the content ablation study, we grouped all codes into six mutually exclusive categories based on ontology prefixes and hierarchical SNOMED parent concepts. Counts were computed across all events from all patients, excluding codes from CARE_SITE and ICDO3, as well as codes with empty descriptions.

**Category**	**Included Codes**	**# Codes**
Demographics	Race/, Gender/, Ethnicity/, SNOMED/3950001 (birth)	18,189 (0.0%)
Visits	Visit/, Medicare Specialty/, CMS Place of Service/	606,798 (1.5%)
Conditions	Cancer Modifier/, OMOP Extension/, Condition Type/, all remaining SNOMED codes	2,995,518 (7.3%)
Medications	RxNorm/, RxNorm Extension/, CVX/, SNOMED descendants of 373873005 (pharmaceutical product), 105590001 (substance)	2,453,197 (5.9%)
Procedures	CPT4/, ICD10PCS/, ICD9Proc/, Domain/, HCPCS/, SNOMED descendants of 71388002 (procedure)	1,840,172 (4.5%)
Lab Results	LOINC/, SNOMED descendants of 108252007 (laboratory procedure), 430925007 (measurement of substance)	33,351,150 (80.8%)
**Total**	*	41,265,024 (100.0%)

Table S9 Performance for All Examples on EHRSHOT for EHR Serialization Experiments. Mean area under the receiver operating characteristic curve (AUROC) performance with approximate 95% confidence intervals of the EHR list serialization used in this work (Full EHR) and different EHR serialization variants. We evaluated a generic and an empty instruction, serializations with specific components removed, and serialization consisting of an individual component.

**Model**	**Operational Outcomes**	**Anticipating Lab Test Results**	**Assignment of New Diagnosis**	**Anticipating Chest X-ray Findings**	**Macro Avg. Across Task Groups**
**Original List Serialization**					
Full EHR	$0.797_{.773-.820}$	$0.842_{.835-.850}$	$0.714_{.672-.757}$	$0.722_{.711-.733}$	$0.769_{.744-.794}$
**Instruction Experiments**					
Generic Instruction	$0.788_{.765-.812}$	$0.772_{.762-.782}$	$0.706_{.665-.747}$	$0.718_{.707-.730}$	$0.746_{.722-.771}$
Empty Instruction	$0.791_{.767-.815}$	$0.756_{.745-.766}$	$0.698_{.651-.745}$	$0.715_{.704-.726}$	$0.740_{.712-.767}$
**Removing Medical Code Categories**					
No Demographics	$0.797_{.774-.820}$	$0.842_{.834-.849}$	$0.708_{.666-.750}$	$0.721_{.710-.732}$	$0.767_{.742-.792}$
No Visits	$0.796_{.773-.819}$	$0.842_{.835-.849}$	$0.717_{.676-.758}$	$0.723_{.712-.734}$	$0.769_{.745-.794}$
No Conditions	$0.797_{.774-.820}$	$0.847_{.840-.854}$	$0.701_{.665-.737}$	$0.716_{.705-.727}$	$0.765_{.743-.788}$
No Medications	$0.798_{.775-.822}$	$0.846_{.838-.853}$	$0.702_{.657-.746}$	$0.722_{.711-.733}$	$0.767_{.741-.793}$
No Procedures	$0.787_{.764-.810}$	$0.847_{.839-.854}$	$0.721_{.678-.764}$	$0.718_{.707-.729}$	$0.768_{.743-.793}$
No Lab Results	$0.798_{.775-.821}$	$0.752_{.741-.763}$	$0.722_{.681-.762}$	$0.711_{.699-.722}$	$0.746_{.721-.770}$
**Individual Medical Code Categories**					
Only Demographics	$0.549_{.518-.580}$	$0.543_{.528-.559}$	$0.606_{.570-.643}$	$0.513_{.494-.531}$	$0.553_{.526-.579}$
Only Visits	$0.591_{.564-.618}$	$0.628_{.615-.642}$	$0.560_{.523-.596}$	$0.613_{.600-.625}$	$0.598_{.574-.622}$
Only Conditions	$0.773_{.750-.797}$	$0.708_{.695-.721}$	$0.693_{.649-.737}$	$0.686_{.673-.698}$	$0.715_{.688-.741}$
Only Medications	$0.777_{.756-.799}$	$0.710_{.698-.722}$	$0.688_{.641-.735}$	$0.657_{.644-.670}$	$0.708_{.681-.735}$
Only Procedures	$0.781_{.756-.807}$	$0.718_{.705-.731}$	$0.672_{.640-.704}$	$0.697_{.685-.708}$	$0.717_{.695-.739}$
Only Lab Results	$0.777_{.755-.799}$	$0.856_{.849-.862}$	$0.665_{.607-.724}$	$0.702_{.691-.713}$	$0.750_{.718-.782}$

Table S10 Performance for All Examples on EHRSHOT Across Context Sizes. Mean area under the receiver operating characteristic curve (AUROC) performance with approximate 95% confidence intervals for different context sizes of the LLM embedding models.

Model	Operational Outcomes	Anticipating Lab Test Results	Assignment of New Diagnosis	Anticipating Chest X-ray Findings	Macro Avg. Across Task Groups
**Qwen3-Emb-8B**					
8,192 context size	0.797 $_{.773-.820}$	0.842 $_{.835-.850}$	0.714 $_{.672-.757}$	0.722 $_{.711-.733}$	0.769 $_{.744-.794}$
4,096 context size	0.800 $_{.778-.823}$	0.850 $_{.843-.857}$	0.711 $_{.666-.757}$	0.727 $_{.716-.737}$	0.772 $_{.746-.798}$
2,048 context size	0.805 $_{.783-.828}$	0.859 $_{.852-.865}$	0.691 $_{.656-.726}$	0.718 $_{.706-.729}$	0.768 $_{.746-.790}$
1,024 context size	0.799 $_{.776-.822}$	0.832 $_{.825-.839}$	0.707 $_{.668-.746}$	0.697 $_{.686-.709}$	0.759 $_{.735-.782}$
512 context size	0.790 $_{.766-.814}$	0.786 $_{.778-.794}$	0.703 $_{.660-.745}$	0.683 $_{.672-.694}$	0.741 $_{.715-.766}$
**Qwen2-Emb-7B**					
8,192 context size	0.772 $_{.749-.796}$	0.746 $_{.735-.757}$	0.744 $_{.705-.784}$	0.685 $_{.671-.699}$	0.737 $_{.712-.761}$
4,096 context size	0.794 $_{.771-.816}$	0.799 $_{.790-.808}$	0.740 $_{.698-.781}$	0.708 $_{.696-.720}$	0.760 $_{.736-.785}$
2,048 context size	0.806 $_{.783-.828}$	0.846 $_{.839-.853}$	0.718 $_{.674-.761}$	0.720 $_{.709-.731}$	0.772 $_{.747-.798}$
1,024 context size	0.797 $_{.776-.817}$	0.833 $_{.826-.841}$	0.709 $_{.665-.752}$	0.700 $_{.689-.711}$	0.760 $_{.735-.785}$
512 context size	0.784 $_{.763-.805}$	0.786 $_{.778-.794}$	0.741 $_{.702-.779}$	0.683 $_{.672-.694}$	0.748 $_{.726-.771}$
**Llama3.1-LLM2Vec-8B**					
8,192 context size	0.763 $_{.738-.787}$	0.726 $_{.714-.738}$	0.727 $_{.688-.766}$	0.686 $_{.673-.699}$	0.725 $_{.701-.750}$
4,096 context size	0.780 $_{.756-.804}$	0.757 $_{.746-.768}$	0.714 $_{.664-.763}$	0.716 $_{.705-.727}$	0.742 $_{.713-.770}$
2,048 context size	0.803 $_{.781-.825}$	0.826 $_{.819-.834}$	0.680 $_{.636-.725}$	0.721 $_{.710-.731}$	0.757 $_{.732-.783}$
1,024 context size	0.790 $_{.766-.813}$	0.843 $_{.836-.850}$	0.696 $_{.654-.738}$	0.700 $_{.689-.710}$	0.757 $_{.732-.782}$
512 context size	0.775 $_{.751-.800}$	0.789 $_{.781-.796}$	0.689 $_{.643-.735}$	0.686 $_{.676-.697}$	0.735 $_{.708-.762}$

Table S11 Performance for All Examples on EHRSHOT Across Time Windows. Mean area under the receiver operating characteristic curve (AUROC) performance with approximate 95% confidence intervals for different time windows of the LLM embedding models and the count-based baseline.

Model	Operational Outcomes	Anticipating Lab Test Results	Assignment of New Diagnosis	Anticipating Chest X-ray Findings	Macro Avg. Across Task Groups
**Qwen3-Emb-8B**					
Full patient history	0.797 $_{.773-.820}$	0.842 $_{.835-.850}$	0.714 $_{.672-.757}$	0.722 $_{.711-.733}$	0.769 $_{.744-.794}$
3 years (1,095 days)	0.798 $_{.775-.821}$	0.842 $_{.835-.849}$	0.717 $_{.674-.759}$	0.723 $_{.712-.734}$	0.770 $_{.745-.795}$
1 year (365 days)	0.802 $_{.780-.824}$	0.843 $_{.836-.850}$	0.720 $_{.677-.762}$	0.723 $_{.712-.734}$	0.772 $_{.747-.797}$
1 month (30 days)	0.814 $_{.794-.835}$	0.840 $_{.832-.847}$	0.695 $_{.651-.739}$	0.717 $_{.707-.727}$	0.767 $_{.742-.792}$
1 week (7 days)	0.813 $_{.792-.834}$	0.832 $_{.824-.839}$	0.683 $_{.645-.722}$	0.702 $_{.692-.712}$	0.758 $_{.735-.780}$
1 day	0.812 $_{.790-.834}$	0.800 $_{.791-.808}$	0.697 $_{.660-.735}$	0.664 $_{.654-.675}$	0.743 $_{.721-.766}$
1 hour	0.764 $_{.740-.788}$	0.677 $_{.668-.686}$	0.627 $_{.585-.668}$	0.602 $_{.591-.612}$	0.667 $_{.642-.692}$
**Qwen2-Emb-7B**					
Full patient history	0.772 $_{.749-.796}$	0.746 $_{.735-.757}$	0.744 $_{.705-.784}$	0.685 $_{.671-.699}$	0.737 $_{.712-.761}$
3 years (1,095 days)	0.773 $_{.750-.796}$	0.754 $_{.743-.765}$	0.725 $_{.681-.770}$	0.688 $_{.675-.701}$	0.735 $_{.709-.761}$
1 year (365 days)	0.781 $_{.758-.803}$	0.766 $_{.756-.776}$	0.724 $_{.685-.763}$	0.696 $_{.683-.709}$	0.742 $_{.718-.766}$
1 month (30 days)	0.810 $_{.789-.831}$	0.789 $_{.780-.798}$	0.715 $_{.679-.752}$	0.711 $_{.701-.722}$	0.756 $_{.734-.779}$
1 week (7 days)	0.812 $_{.791-.833}$	0.801 $_{.792-.809}$	0.700 $_{.658-.741}$	0.702 $_{.692-.712}$	0.754 $_{.729-.778}$
1 day	0.817 $_{.797-.837}$	0.796 $_{.788-.804}$	0.720 $_{.669-.771}$	0.663 $_{.653-.673}$	0.749 $_{.721-.777}$
1 hour	0.771 $_{.749-.794}$	0.674 $_{.665-.682}$	0.599 $_{.558-.640}$	0.600 $_{.589-.610}$	0.661 $_{.637-.685}$
**Llama3.1-LLM2Vec-8B**					
Full patient history	0.763 $_{.738-.787}$	0.726 $_{.714-.738}$	0.727 $_{.688-.766}$	0.686 $_{.673-.699}$	0.725 $_{.701-.750}$
3 years (1,095 days)	0.763 $_{.739-.787}$	0.733 $_{.721-.744}$	0.696 $_{.662-.730}$	0.694 $_{.682-.705}$	0.721 $_{.699-.744}$
1 year (365 days)	0.776 $_{.753-.800}$	0.742 $_{.731-.754}$	0.706 $_{.657-.756}$	0.705 $_{.694-.717}$	0.733 $_{.704-.761}$
1 month (30 days)	0.802 $_{.780-.824}$	0.770 $_{.760-.780}$	0.696 $_{.642-.751}$	0.718 $_{.707-.728}$	0.747 $_{.716-.777}$
1 week (7 days)	0.816 $_{.795-.836}$	0.782 $_{.773-.791}$	0.687 $_{.634-.740}$	0.702 $_{.691-.712}$	0.747 $_{.718-.776}$
1 day	0.811 $_{.790-.833}$	0.786 $_{.777-.794}$	0.680 $_{.626-.734}$	0.666 $_{.656-.676}$	0.736 $_{.706-.766}$
1 hour	0.766 $_{.743-.790}$	0.673 $_{.664-.681}$	0.581 $_{.538-.625}$	0.595 $_{.585-.606}$	0.654 $_{.628-.680}$
**Count-based + GBM**					
Full patient history	0.824 $_{.804-.844}$	0.841 $_{.833-.849}$	0.758 $_{.724-.793}$	0.686 $_{.674-.699}$	0.777 $_{.756-.799}$
3 years (1,095 days)	0.819 $_{.799-.839}$	0.841 $_{.833-.850}$	0.740 $_{.691-.790}$	0.688 $_{.676-.700}$	0.772 $_{.745-.800}$
1 year (365 days)	0.824 $_{.803-.844}$	0.841 $_{.833-.849}$	0.749 $_{.705-.794}$	0.679 $_{.666-.692}$	0.773 $_{.748-.799}$
1 month (30 days)	0.824 $_{.805-.844}$	0.836 $_{.828-.844}$	0.707 $_{.669-.746}$	0.688 $_{.677-.700}$	0.764 $_{.741-.787}$
1 week (7 days)	0.814 $_{.793-.835}$	0.842 $_{.835-.850}$	0.693 $_{.653-.734}$	0.686 $_{.674-.697}$	0.759 $_{.735-.782}$
1 day	0.820 $_{.799-.841}$	0.830 $_{.823-.837}$	0.702 $_{.660-.745}$	0.650 $_{.638-.662}$	0.751 $_{.726-.775}$
1 hour	0.742 $_{.717-.767}$	0.670 $_{.662-.679}$	0.616 $_{.569-.664}$	0.572 $_{.559-.586}$	0.650 $_{.622-.678}$

Table S12 Encoding Times for Models on EHRSHOT. Time required to encode EHR entries for EHRSHOT experiments on an 8-GPU H200 cluster. LLM embedding models and encoder language models use EHR list serialization with inputs of up to 8,192 tokens. Encoder language models process the input in separate 512-token chunks. Differences in multi-GPU optimization across models may affect runtime. We attempted to optimize each model for the given GPU cluster.

**Model**	**Encoding Time**	**Macro Avg. Across Task Groups**
**Baselines** [45]		
CLMBR-T-Base	6:04	0.769 $_{.746-.792}$
**LLM Embedding Models**		
Qwen3-Emb-8B	21:48:56	0.769 $_{.744-.794}$
Qwen3-Emb-4B	12:20:28	0.759 $_{.730-.787}$
Qwen3-Emb-0.6B	5:14:07	0.727 $_{.697-.758}$
Qwen2-Emb-7B	12:47:18	0.737 $_{.712-.761}$
Qwen2-Emb-1.5B	5:23:55	0.710 $_{.683-.738}$
Llama3.1-LLM2Vec-8B	21:39:06	0.725 $_{.701-.750}$
**Encoder Language Models**		
DeBERTaV3 large	7:00:21	0.692 $_{.664-.720}$
DeBERTaV3 base	2:49:35	0.685 $_{.660-.709}$
BERT large	5:31:18	0.694 $_{.667-.722}$
BERT base	2:21:35	0.701 $_{.674-.727}$

Table S13 Hyperparameter Tuning Results for Fine-Tuning Experiments. Mean validation AUROC across the tuning sweep for the operational outcomes and assignment of new diagnoses tasks at $k \in \{8, 16\}$. Rows marked with a * denote the selected configuration for each model family.

Config ID	LR	r	Dropout	$k = 8$ Val. AUROC	$k = 16$ Val. AUROC	Macro Avg.
**Encoder Models**						
enc_lr5e5_r8_d000	5e-5	8	0.00	0.667	0.688	0.677
enc_lr5e5_r8_d005	5e-5	8	0.05	0.641	0.696	0.668
enc_lr5e5_r8_d010*	5e-5	8	0.10	0.686	0.720	0.703
enc_lr5e5_r16_d000	5e-5	16	0.00	0.616	0.651	0.634
enc_lr5e5_r16_d005	5e-5	16	0.05	0.606	0.686	0.646
enc_lr5e5_r16_d010	5e-5	16	0.10	0.578	0.617	0.597
enc_lr5e5_r64_d000	5e-5	64	0.00	0.639	0.696	0.667
enc_lr5e5_r64_d005	5e-5	64	0.05	0.668	0.677	0.672
enc_lr5e5_r64_d010	5e-5	64	0.10	0.658	0.634	0.646
enc_lr1e4_r8_d000	1e-4	8	0.00	0.691	0.630	0.660
enc_lr1e4_r8_d005	1e-4	8	0.05	0.592	0.657	0.625
enc_lr1e4_r8_d010	1e-4	8	0.10	0.668	0.681	0.675
enc_lr1e4_r16_d000	1e-4	16	0.00	0.615	0.660	0.637
enc_lr1e4_r16_d005	1e-4	16	0.05	0.670	0.630	0.650
enc_lr1e4_r16_d010	1e-4	16	0.10	0.641	0.653	0.647
enc_lr1e4_r64_d000	1e-4	64	0.00	0.623	0.689	0.656
enc_lr1e4_r64_d005	1e-4	64	0.05	0.584	0.670	0.627
enc_lr1e4_r64_d010	1e-4	64	0.10	0.587	0.686	0.637
enc_lr2e4_r8_d000	2e-4	8	0.00	0.616	0.698	0.657
enc_lr2e4_r8_d005	2e-4	8	0.05	0.655	0.655	0.655
enc_lr2e4_r8_d010	2e-4	8	0.10	0.616	0.686	0.651
enc_lr2e4_r16_d000	2e-4	16	0.00	0.588	0.632	0.610
enc_lr2e4_r16_d005	2e-4	16	0.05	0.596	0.703	0.650
enc_lr2e4_r16_d010	2e-4	16	0.10	0.705	0.681	0.693
enc_lr2e4_r64_d000	2e-4	64	0.00	0.670	0.703	0.687
enc_lr2e4_r64_d005	2e-4	64	0.05	0.627	0.630	0.628
enc_lr2e4_r64_d010	2e-4	64	0.10	0.588	0.663	0.625
**Decoder Models**						
dec_lr5e5_r8_d000	5e-5	8	0.00	0.614	0.606	0.610
dec_lr5e5_r8_d005	5e-5	8	0.05	0.616	0.611	0.614
dec_lr5e5_r8_d010	5e-5	8	0.10	0.624	0.615	0.620
dec_lr5e5_r16_d000	5e-5	16	0.00	0.609	0.622	0.616
dec_lr5e5_r16_d005	5e-5	16	0.05	0.615	0.606	0.610
dec_lr5e5_r16_d010	5e-5	16	0.10	0.615	0.633	0.624
dec_lr5e5_r64_d000	5e-5	64	0.00	0.609	0.609	0.609
dec_lr5e5_r64_d005	5e-5	64	0.05	0.629	0.624	0.627
dec_lr5e5_r64_d010	5e-5	64	0.10	0.616	0.625	0.621
dec_lr1e4_r8_d000	1e-4	8	0.00	0.606	0.629	0.617
dec_lr1e4_r8_d005	1e-4	8	0.05	0.619	0.625	0.622
dec_lr1e4_r8_d010	1e-4	8	0.10	0.610	0.613	0.612
dec_lr1e4_r16_d000	1e-4	16	0.00	0.641	0.619	0.630
dec_lr1e4_r16_d005	1e-4	16	0.05	0.627	0.628	0.627
dec_lr1e4_r16_d010	1e-4	16	0.10	0.621	0.605	0.613
dec_lr1e4_r64_d000	1e-4	64	0.00	0.623	0.624	0.623
dec_lr1e4_r64_d005	1e-4	64	0.05	0.628	0.615	0.622
dec_lr1e4_r64_d010	1e-4	64	0.10	0.625	0.628	0.627
dec_lr2e4_r8_d000	2e-4	8	0.00	0.632	0.651	0.642
dec_lr2e4_r8_d005*	2e-4	8	0.05	0.706	0.637	0.671
dec_lr2e4_r8_d010	2e-4	8	0.10	0.652	0.638	0.645
dec_lr2e4_r16_d000	2e-4	16	0.00	0.657	0.645	0.651
dec_lr2e4_r16_d005	2e-4	16	0.05	0.643	0.621	0.632
dec_lr2e4_r16_d010	2e-4	16	0.10	0.668	0.632	0.650
dec_lr2e4_r64_d000	2e-4	64	0.00	0.674	0.647	0.660
dec_lr2e4_r64_d005	2e-4	64	0.05	0.651	0.619	0.635
dec_lr2e4_r64_d010	2e-4	64	0.10	0.646	0.636	0.641

1.3 Additional Performance Results on EHRSHOT

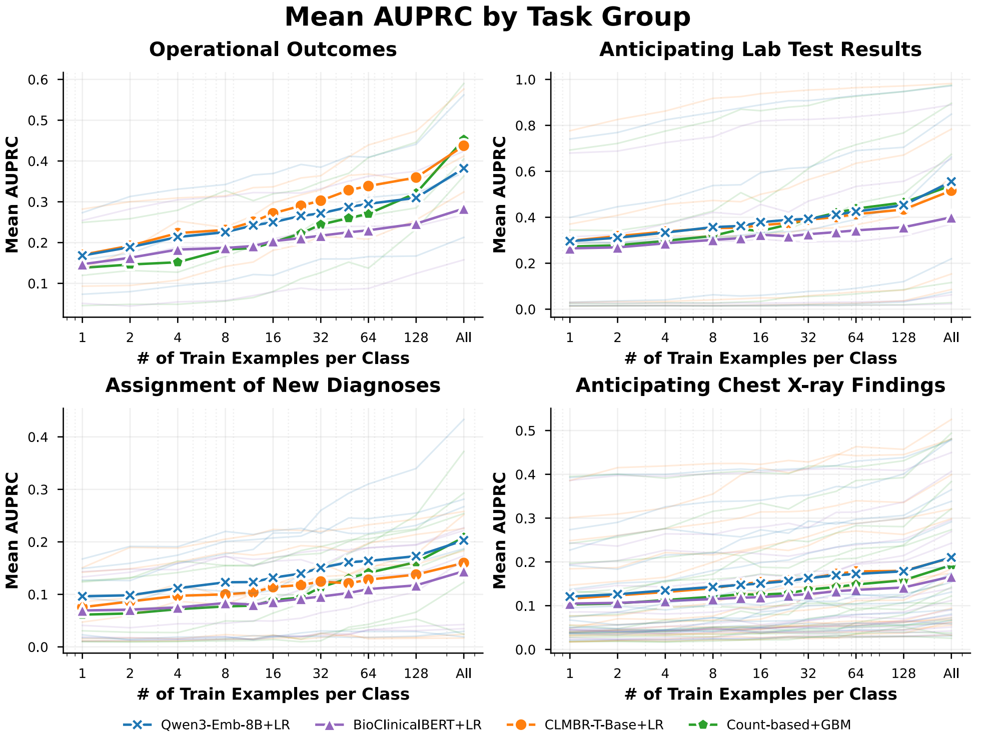

Figure S1 Few-Shot AUPRC Performance on EHRSHOT. Mean area under the precision-recall curve (AUPRC) performance across subtasks for four task groups (bold). Blurred lines show averages across five bootstrapped runs using different seeds [45].

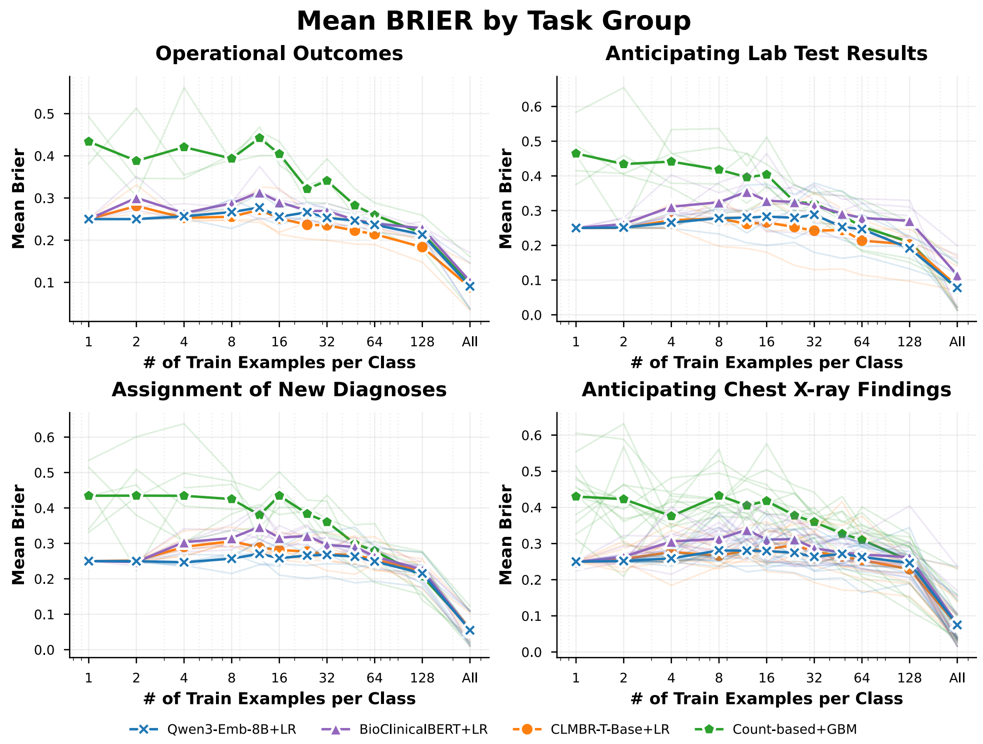

Figure S2 Few-Shot Brier Score on EHRSHOT. Mean Brier score across subtasks for four task groups (bold). Blurred lines show averages across five bootstrapped runs using different seeds [45].

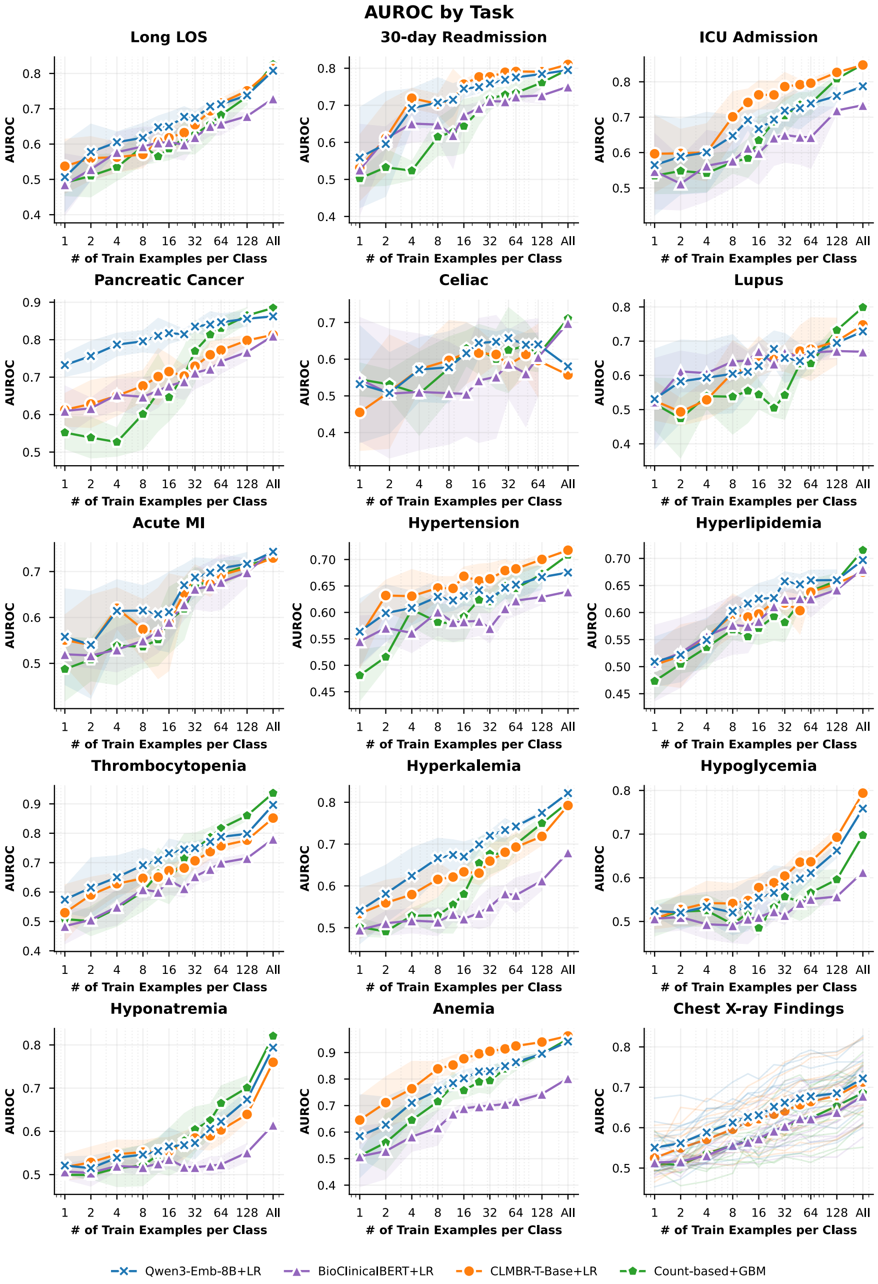

Figure S3 Task-Specific AUROC Performance on EHRSHOT. Area under the receiver operating characteristic curve (AUROC) performance with standard deviation across five few-shot replicates for all 15 prediction tasks [45].

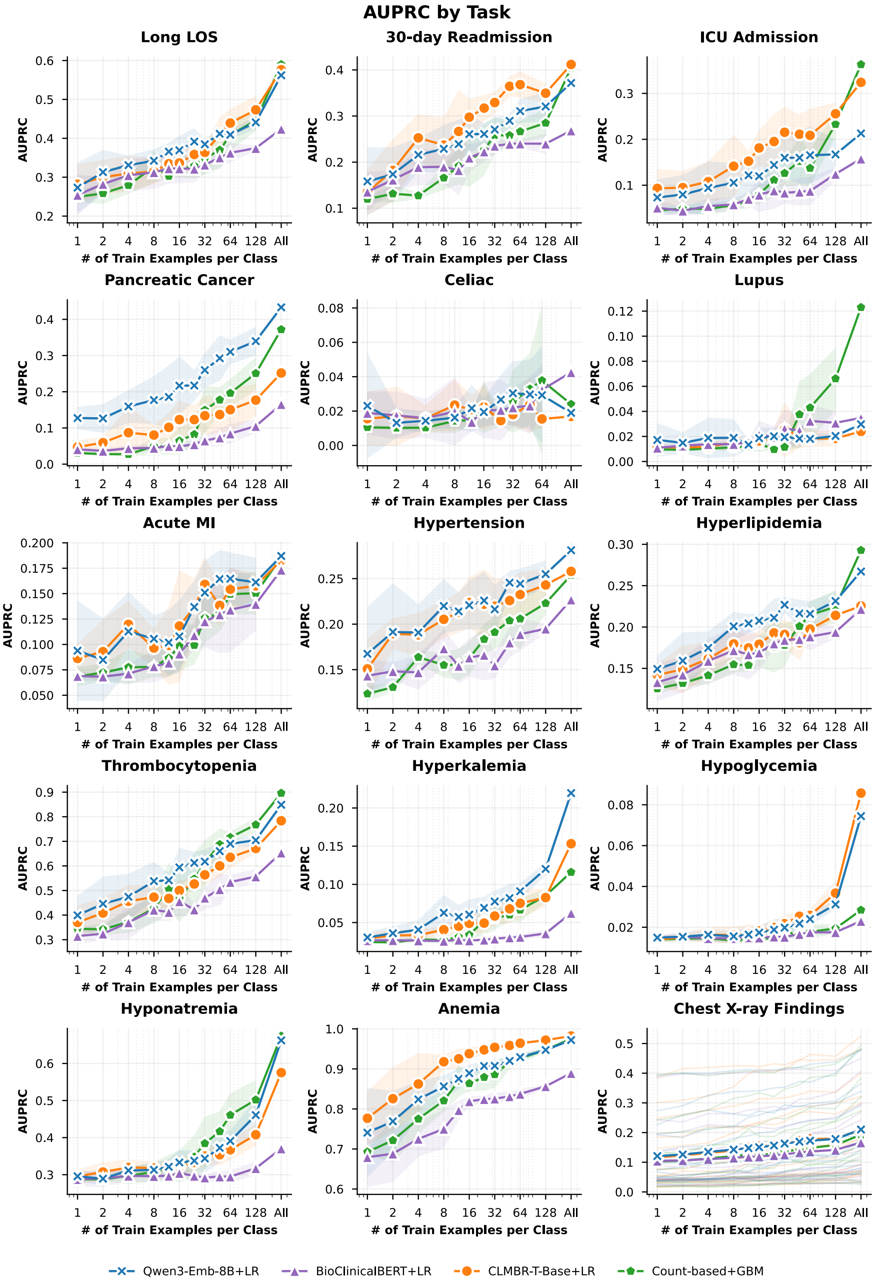

Figure S4 Task-Specific AUPRC Performance on EHRSHOT. Area under the precision-recall curve (AUPRC) performance with standard deviation across five few-shot replicates for all 15 prediction tasks [45].

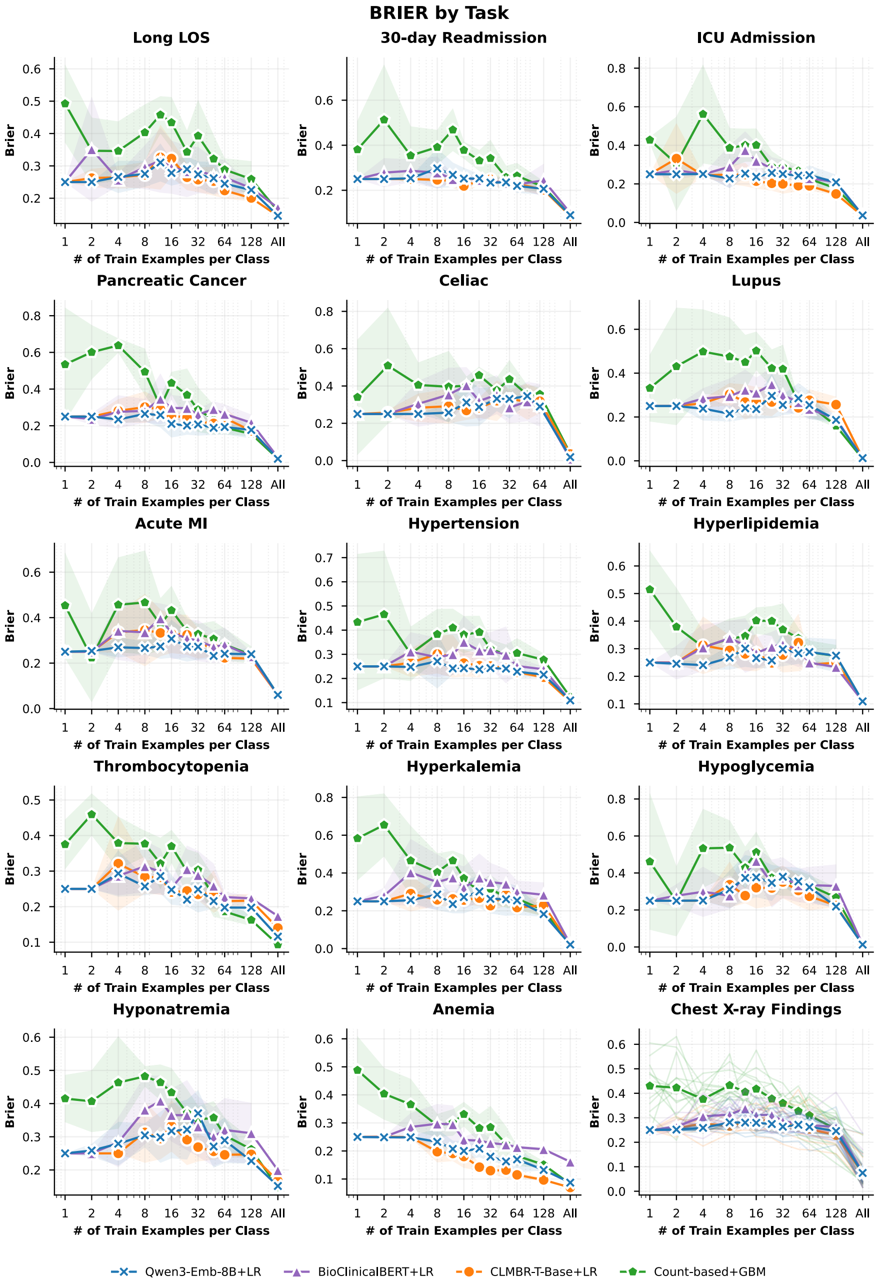

Figure S5 Task-Specific Brier Score on EHRSHOT. Brier score with standard deviation across five few-shot replicates for all 15 prediction tasks [45].

1.4 Additional Performance Results on UK Biobank

Table S14 Performance for All Examples on UKB. Mean area under the receiver operating characteristic curve (AUROC) performance with approximate 95% confidence intervals for three task groups. The assignment of new diagnoses prediction is based on the mean of all 23 provided diseases. The macro-averaged performance across all task groups is given in the right-most column. All LLM embedding models use a context size of 8,192 tokens.

Model	Mortality prediction	Operational Outcomes (Hospitalization)	Assignment of New Diagnoses	Macro Avg. Across Task Groups
**Baselines** [45]				
CLMBR-T-Base	0.801 $_{.772-.830}$	0.689 $_{.685-.693}$	0.719 $_{.708-.731}$	0.736 $_{.726-.747}$
Count-based + GBM	0.780 $_{.749-.810}$	0.709 $_{.705-.712}$	0.634 $_{.622-.646}$	0.708 $_{.697-.719}$
**LLM Embedding Model**				
Qwen3-Emb-8B	0.811 $_{.781-.840}$	0.698 $_{.694-.702}$	0.743 $_{.731-.755}$	0.751 $_{.740-.761}$
Qwen2-Emb-7B	0.804 $_{.774-.835}$	0.695 $_{.691-.699}$	0.747 $_{.732-.762}$	0.749 $_{.737-.760}$
Llama3.1-LLM2Vec-8B	0.796 $_{.766-.827}$	0.690 $_{.686-.694}$	0.731 $_{.717-.746}$	0.739 $_{.728-.751}$
**Sensitivity Analysis Restricted to CLMBR Codes**				
Qwen3-Emb-8B CLMBR-T-Base codes	0.806 $_{.775-.836}$	0.687 $_{.683-.691}$	0.736 $_{.723-.749}$	0.743 $_{.732-.754}$
**Encoder Language Models with Chunked Inputs**				
BioClinicalBERT	0.778 $_{.746-.811}$	0.675 $_{.671-.678}$	0.705 $_{.693-.717}$	0.719 $_{.708-.731}$

Table S15 Per-task ΔAUROC (Qwen3-Emb-8B minus baseline) on UKB. Cells report the AUROC difference (ΔAUROC), 95% bootstrap confidence intervals, and Holm-adjusted p-values obtained from paired patient-level bootstrap tests with 10,000 resamples. Positive values indicate better performance of Qwen3-Emb-8B. Multiple testing was controlled separately for each shot setting ($k = 8$, $k = 64$, and all training data) using Holm's procedure across all tasks and baseline comparisons (75/60 tests per setting). Bold indicates statistically significant differences ($p_{\text{adj}} < 0.05$).

Task	CLMBR-T-Base	BioClinicalBERT	Count-based Model
**8-shot (8 positive / 8 negative examples)**			
Hospitalization	**+0.044** [+0.039, +0.049] (0.015)	**+0.042** [+0.038, +0.047] (0.015)	**−0.032** [−0.036, −0.027] (0.015)
Death	+0.032 [+0.000, +0.065] (1.000)	**+0.083** [+0.046, +0.121] (0.015)	**+0.076** [+0.036, +0.117] (0.015)
Hypertension	+0.010 [−0.004, +0.024] (1.000)	**+0.043** [+0.029, +0.058] (0.015)	+0.006 [−0.008, +0.020] (1.000)
Diabetes Mellitus	+0.016 [−0.014, +0.045] (1.000)	−0.044 [−0.073, −0.015] (0.312)	+0.012 [−0.019, +0.042] (1.000)
Atrial Fibrillation	−0.023 [−0.069, +0.023] (1.000)	−0.004 [−0.040, +0.033] (1.000)	−0.013 [−0.048, +0.024] (1.000)
Pneumonia	−0.045 [−0.084, −0.006] (1.000)	+0.029 [−0.005, +0.063] (1.000)	−0.028 [−0.069, +0.012] (1.000)
COPD	+0.021 [−0.010, +0.052] (1.000)	**+0.072** [+0.040, +0.105] (0.015)	−0.006 [−0.038, +0.027] (1.000)
Chronic Kidney Disease	+0.046 [+0.013, +0.078] (0.399)	−0.020 [−0.052, +0.012] (1.000)	−0.021 [−0.051, +0.009] (1.000)
Ischemic Heart Disease	+0.036 [+0.011, +0.061] (0.360)	−0.007 [−0.030, +0.016] (1.000)	−0.029 [−0.056, −0.002] (1.000)
Myocardial Infarction	+0.052 [−0.001, +0.104] (1.000)	**+0.114** [+0.061, +0.166] (0.015)	+0.030 [−0.019, +0.078] (1.000)
Cerebral Infarction	+0.114 [+0.041, +0.182] (0.126)	−0.013 [−0.070, +0.045] (1.000)	−0.050 [−0.101, −0.002] (1.000)
Heart Failure	−0.079 [−0.133, −0.025] (0.319)	−0.040 [−0.100, +0.019] (1.000)	−0.031 [−0.085, +0.023] (1.000)
Cardiac Arrest	+0.010 [−0.105, +0.123] (1.000)	+0.022 [−0.073, +0.112] (1.000)	+0.144 [+0.029, +0.264] (0.795)
Abdominal Aortic Aneurysm	+0.036 [−0.031, +0.100] (1.000)	+0.013 [−0.034, +0.062] (1.000)	+0.033 [−0.052, +0.118] (1.000)
Pulmonary Embolism	−0.113 [−0.207, −0.017] (1.000)	+0.122 [+0.039, +0.203] (0.232)	−0.059 [−0.153, +0.031] (1.000)
Aortic Stenosis	−0.011 [−0.082, +0.059] (1.000)	+0.033 [−0.078, +0.148] (1.000)	−0.018 [−0.144, +0.114] (1.000)
Mitral Valve Insufficiency	+0.072 [−0.003, +0.143] (1.000)	−0.049 [−0.124, +0.027] (1.000)	+0.078 [−0.015, +0.173] (1.000)
Endocarditis	+0.042 [−0.061, +0.142] (1.000)	−0.011 [−0.102, +0.078] (1.000)	+0.007 [−0.095, +0.108] (1.000)
Rheumatic Fever	+0.080 [+0.016, +0.143] (0.792)	+0.085 [+0.021, +0.152] (0.582)	**+0.206** [+0.110, +0.302] (0.015)
Anemia	+0.021 [−0.005, +0.047] (1.000)	+0.017 [−0.009, +0.043] (1.000)	+0.044 [+0.017, +0.071] (0.126)
Back Pain	**+0.126** [+0.105, +0.146] (0.015)	**+0.052** [+0.038, +0.066] (0.015)	−0.004 [−0.017, +0.010] (1.000)
Parkinson's Disease	−0.075 [−0.215, +0.069] (1.000)	+0.058 [−0.075, +0.184] (1.000)	−0.077 [−0.208, +0.062] (1.000)
Rheumatoid Arthritis	−0.059 [−0.133, +0.016] (1.000)	−0.007 [−0.082, +0.069] (1.000)	−0.006 [−0.066, +0.055] (1.000)
Psoriasis	+0.079 [+0.015, +0.143] (0.792)	+0.043 [−0.004, +0.091] (1.000)	+0.027 [−0.020, +0.073] (1.000)
Suicide Ideation / Self Harm	+0.041 [−0.027, +0.108] (1.000)	+0.117 [+0.047, +0.191] (0.090)	+0.072 [−0.007, +0.154] (1.000)
**64-shot (64 positive / 64 negative examples)**			
Hospitalization	**+0.023** [+0.018, +0.028] (0.012)	−0.002 [−0.006, +0.002] (1.000)	+0.002 [−0.003, +0.007] (1.000)
Death	+0.017 [−0.008, +0.040] (1.000)	+0.029 [+0.003, +0.055] (1.000)	+0.006 [−0.019, +0.031] (1.000)
Hypertension	+0.007 [−0.006, +0.020] (1.000)	**+0.038** [+0.026, +0.050] (0.012)	−0.003 [−0.016, +0.010] (1.000)
Diabetes Mellitus	**+0.068** [+0.044, +0.092] (0.012)	+0.031 [+0.012, +0.051] (0.150)	+0.008 [−0.011, +0.027] (1.000)
Atrial Fibrillation	+0.004 [−0.031, +0.041] (1.000)	+0.036 [+0.007, +0.067] (0.475)	+0.007 [−0.018, +0.032] (1.000)
Pneumonia	−0.031 [−0.065, +0.003] (1.000)	−0.021 [−0.056, +0.014] (1.000)	−0.063 [−0.099, −0.027] (0.071)
COPD	+0.029 [+0.005, +0.054] (0.697)	**+0.079** [+0.052, +0.106] (0.012)	−0.016 [−0.038, +0.006] (1.000)
Chronic Kidney Disease	+0.017 [−0.003, +0.038] (1.000)	+0.022 [+0.006, +0.037] (0.202)	−0.005 [−0.021, +0.011] (1.000)
Ischemic Heart Disease	−0.018 [−0.040, +0.003] (1.000)	+0.014 [−0.005, +0.033] (1.000)	−0.033 [−0.054, −0.011] (0.125)
Myocardial Infarction	−0.027 [−0.063, +0.009] (1.000)	+0.068 [+0.028, +0.108] (0.064)	−0.022 [−0.057, +0.013] (1.000)
Cerebral Infarction	+0.038 [−0.010, +0.085] (1.000)	+0.034 [−0.001, +0.069] (1.000)	−0.040 [−0.096, +0.015] (1.000)
Heart Failure	+0.009 [−0.020, +0.038] (1.000)	+0.037 [+0.008, +0.066] (0.475)	+0.005 [−0.029, +0.041] (1.000)
Cardiac Arrest	—	—	—
Abdominal Aortic Aneurysm	—	—	—
Pulmonary Embolism	−0.047 [−0.122, +0.029] (1.000)	+0.090 [+0.023, +0.157] (0.360)	+0.017 [−0.063, +0.099] (1.000)
Aortic Stenosis	—	—	—
Mitral Valve Insufficiency	+0.053 [−0.000, +0.107] (1.000)	+0.079 [+0.028, +0.132] (0.071)	**+0.132** [+0.067, +0.200] (0.012)
Endocarditis	—	—	—
Rheumatic Fever	+0.031 [−0.027, +0.090] (1.000)	−0.011 [−0.074, +0.049] (1.000)	+0.004 [−0.047, +0.054] (1.000)
Anemia	+0.002 [−0.023, +0.027] (1.000)	+0.005 [−0.021, +0.032] (1.000)	−0.043 [−0.067, −0.017] (0.078)
Back Pain	**+0.041** [+0.030, +0.052] (0.012)	−0.001 [−0.011, +0.008] (1.000)	−0.003 [−0.013, +0.006] (1.000)
Parkinson's Disease	—	—	—
Rheumatoid Arthritis	+0.009 [−0.047, +0.068] (1.000)	+0.041 [−0.012, +0.094] (1.000)	−0.005 [−0.058, +0.048] (1.000)
Psoriasis	+0.042 [+0.002, +0.082] (1.000)	+0.033 [+0.001, +0.064] (1.000)	+0.010 [−0.024, +0.044] (1.000)
Suicide Ideation / Self Harm	**+0.116** [+0.052, +0.179] (0.043)	+0.103 [+0.040, +0.170] (0.064)	+0.047 [−0.005, +0.103] (1.000)

Table S16 Per-task ΔAUROC (Qwen3-Emb-8B minus baseline) on UKB (continued).

Task	CLMBR-T-Base	BioClinicalBERT	Count-based Model
**All training data**			
Hospitalization	**+0.009**$_{[+0.007,+0.012]}$ (0.015)	**+0.023**$_{[+0.021,+0.026]}$ (0.015)	**−0.011**$_{[-0.013,-0.008]}$ (0.015)
Death	+0.009$_{[-0.009,+0.029]}$ (1.000)	+0.032$_{[+0.010,+0.056]}$ (0.263)	+0.016$_{[-0.010,+0.041]}$ (1.000)
Hypertension	**+0.019**$_{[+0.009,+0.028]}$ (0.015)	**+0.032**$_{[+0.024,+0.040]}$ (0.015)	**−0.022**$_{[-0.031,-0.012]}$ (0.015)
Diabetes Mellitus	**+0.046**$_{[+0.027,+0.065]}$ (0.015)	**+0.034**$_{[+0.017,+0.052]}$ (0.021)	−0.018$_{[-0.037,+0.001]}$ (1.000)
Atrial Fibrillation	+0.038$_{[-0.001,+0.080]}$ (1.000)	+0.030$_{[+0.001,+0.060]}$ (1.000)	**+0.358**$_{[+0.284,+0.432]}$ (0.015)
Pneumonia	−0.019$_{[-0.044,+0.005]}$ (1.000)	+0.036$_{[+0.011,+0.060]}$ (0.250)	+0.005$_{[-0.022,+0.032]}$ (1.000)
COPD	**+0.045**$_{[+0.022,+0.068]}$ (0.015)	**+0.067**$_{[+0.046,+0.089]}$ (0.015)	−0.019$_{[-0.041,+0.003]}$ (1.000)
Chronic Kidney Disease	+0.026$_{[+0.008,+0.045]}$ (0.270)	**+0.041**$_{[+0.022,+0.059]}$ (0.015)	**+0.045**$_{[+0.024,+0.067]}$ (0.015)
Ischemic Heart Disease	−0.009$_{[-0.024,+0.006]}$ (1.000)	**+0.026**$_{[+0.011,+0.040]}$ (0.041)	−0.014$_{[-0.031,+0.003]}$ (1.000)
Myocardial Infarction	−0.006$_{[-0.034,+0.021]}$ (1.000)	+0.037$_{[+0.011,+0.063]}$ (0.273)	−0.014$_{[-0.049,+0.020]}$ (1.000)
Cerebral Infarction	+0.007$_{[-0.039,+0.051]}$ (1.000)	+0.034$_{[-0.002,+0.071]}$ (1.000)	+0.073$_{[-0.013,+0.159]}$ (1.000)
Heart Failure	+0.014$_{[-0.014,+0.042]}$ (1.000)	+0.040$_{[+0.013,+0.067]}$ (0.273)	**+0.166**$_{[+0.112,+0.222]}$ (0.015)
Cardiac Arrest	+0.003$_{[-0.088,+0.094]}$ (1.000)	+0.040$_{[-0.039,+0.120]}$ (1.000)	+0.196$_{[+0.068,+0.326]}$ (0.167)
Abdominal Aortic Aneurysm	+0.067$_{[+0.005,+0.126]}$ (1.000)	+0.039$_{[-0.021,+0.100]}$ (1.000)	**+0.346**$_{[+0.236,+0.449]}$ (0.015)
Pulmonary Embolism	−0.002$_{[-0.073,+0.069]}$ (1.000)	+0.093$_{[+0.027,+0.159]}$ (0.267)	+0.109$_{[+0.031,+0.192]}$ (0.311)
Aortic Stenosis	+0.046$_{[-0.007,+0.099]}$ (1.000)	+0.001$_{[-0.070,+0.075]}$ (1.000)	+0.228$_{[+0.097,+0.353]}$ (0.080)
Mitral Valve Insufficiency	+0.042$_{[-0.009,+0.096]}$ (1.000)	+0.069$_{[+0.018,+0.123]}$ (0.435)	**+0.300**$_{[+0.232,+0.363]}$ (0.015)
Endocarditis	+0.038$_{[-0.026,+0.101]}$ (1.000)	−0.040$_{[-0.127,+0.041]}$ (1.000)	+0.155$_{[+0.016,+0.301]}$ (1.000)
Rheumatic Fever	+0.044$_{[-0.011,+0.100]}$ (1.000)	+0.010$_{[-0.044,+0.063]}$ (1.000)	**+0.318**$_{[+0.248,+0.382]}$ (0.015)
Anemia	**+0.038**$_{[+0.019,+0.058]}$ (0.015)	**+0.047**$_{[+0.028,+0.066]}$ (0.015)	+0.010$_{[-0.010,+0.029]}$ (1.000)
Back Pain	**+0.055**$_{[+0.046,+0.063]}$ (0.015)	+0.008$_{[+0.001,+0.015]}$ (1.000)	**−0.016**$_{[-0.023,-0.009]}$ (0.015)
Parkinson's Disease	−0.051$_{[-0.144,+0.039]}$ (1.000)	**+0.131**$_{[+0.060,+0.204]}$ (0.031)	+0.135$_{[-0.019,+0.277]}$ (1.000)
Rheumatoid Arthritis	+0.020$_{[-0.038,+0.081]}$ (1.000)	+0.057$_{[-0.004,+0.120]}$ (1.000)	+0.072$_{[-0.005,+0.152]}$ (1.000)
Psoriasis	+0.031$_{[+0.001,+0.061]}$ (1.000)	+0.001$_{[-0.027,+0.028]}$ (1.000)	**+0.127**$_{[+0.074,+0.179]}$ (0.015)
Suicide Ideation / Self Harm	+0.058$_{[+0.008,+0.107]}$ (1.000)	+0.048$_{[-0.013,+0.110]}$ (1.000)	**+0.300**$_{[+0.152,+0.445]}$ (0.015)

Table S17 Per-task ΔAUROC (Qwen3-Emb-8B restricted to CLMBR-T-Base codes minus CLMBR-T-Base) on UKB. Cells report the AUROC difference (ΔAUROC), 95% bootstrap confidence intervals, and Holm-adjusted p-values obtained from paired patient-level bootstrap tests with 10,000 resamples. Positive values indicate better performance of Qwen3-Emb-8B. Multiple testing was controlled separately for each shot setting ($k = 8$, $k = 64$, and all training data) using Holm's procedure across all tasks and baseline comparisons (25/20 tests per setting). Bold indicates statistically significant differences ($p_{\text{adj}} < 0.05$).

Task	8-shot	64-shot	All training data
Hospitalization	**+0.048** [+0.043, +0.053] (0.005)	−0.007 [−0.012, −0.002] (0.116)	−0.002 [−0.004, +0.001] (1.000)
Death	**−0.156** [−0.200, −0.111] (0.005)	+0.005 [−0.021, +0.033] (1.000)	+0.004 [−0.015, +0.024] (1.000)
Hypertension	+0.007 [−0.007, +0.020] (1.000)	−0.013 [−0.026, +0.000] (0.780)	+0.003 [−0.006, +0.012] (1.000)
Diabetes Mellitus	**−0.060** [−0.094, −0.026] (0.008)	+0.031 [+0.006, +0.057] (0.266)	**+0.035** [+0.016, +0.054] (0.014)
Atrial Fibrillation	−0.008 [−0.055, +0.041] (1.000)	−0.026 [−0.064, +0.013] (1.000)	+0.006 [−0.033, +0.047] (1.000)
Pneumonia	−0.065 [−0.109, −0.020] (0.094)	+0.006 [−0.029, +0.041] (1.000)	−0.036 [−0.064, −0.008] (0.232)
COPD	+0.022 [−0.009, +0.055] (1.000)	+0.003 [−0.024, +0.028] (1.000)	+0.024 [+0.001, +0.046] (0.788)
Chronic Kidney Disease	**+0.067** [+0.040, +0.094] (0.005)	+0.006 [−0.013, +0.026] (1.000)	+0.002 [−0.017, +0.021] (1.000)
Ischemic Heart Disease	**+0.102** [+0.077, +0.127] (0.005)	+0.018 [−0.003, +0.038] (1.000)	+0.002 [−0.013, +0.016] (1.000)
Myocardial Infarction	**+0.082** [+0.031, +0.133] (0.040)	+0.015 [−0.016, +0.046] (1.000)	+0.030 [+0.004, +0.056] (0.502)
Cerebral Infarction	+0.081 [+0.017, +0.143] (0.194)	+0.018 [−0.030, +0.066] (1.000)	+0.013 [−0.026, +0.049] (1.000)
Heart Failure	−0.037 [−0.086, +0.013] (1.000)	−0.005 [−0.036, +0.024] (1.000)	+0.001 [−0.028, +0.029] (1.000)
Cardiac Arrest	+0.013 [−0.105, +0.124] (1.000)	—	−0.044 [−0.148, +0.055] (1.000)
Abdominal Aortic Aneurysm	+0.060 [+0.010, +0.112] (0.352)	—	+0.083 [+0.021, +0.140] (0.202)
Pulmonary Embolism	−0.112 [−0.207, −0.015] (0.402)	−0.056 [−0.125, +0.013] (1.000)	−0.039 [−0.103, +0.026] (1.000)
Aortic Stenosis	+0.049 [−0.030, +0.131] (1.000)	—	+0.065 [−0.009, +0.137] (1.000)
Mitral Valve Insufficiency	+0.077 [−0.005, +0.155] (0.845)	+0.031 [−0.023, +0.085] (1.000)	+0.016 [−0.033, +0.065] (1.000)
Endocarditis	+0.085 [+0.003, +0.170] (0.619)	—	+0.075 [−0.020, +0.164] (1.000)
Rheumatic Fever	−0.060 [−0.127, +0.008] (0.994)	+0.022 [−0.040, +0.084] (1.000)	−0.030 [−0.091, +0.031] (1.000)
Anemia	−0.003 [−0.032, +0.027] (1.000)	+0.014 [−0.011, +0.039] (1.000)	+0.027 [+0.010, +0.045] (0.070)
Back Pain	−0.010 [−0.030, +0.010] (1.000)	**+0.024** [+0.013, +0.035] (0.004)	**+0.040** [+0.032, +0.048] (0.005)
Parkinson's Disease	−0.059 [−0.176, +0.067] (1.000)	—	−0.029 [−0.136, +0.072] (1.000)
Rheumatoid Arthritis	−0.044 [−0.115, +0.026] (1.000)	−0.044 [−0.104, +0.017] (1.000)	+0.034 [−0.024, +0.096] (1.000)
Psoriasis	**+0.092** [+0.029, +0.154] (0.042)	**+0.062** [+0.024, +0.100] (0.034)	+0.049 [+0.019, +0.080] (0.055)
Suicide Ideation / Self Harm	−0.046 [−0.134, +0.042] (1.000)	**+0.109** [+0.043, +0.178] (0.036)	+0.052 [−0.001, +0.105] (0.887)

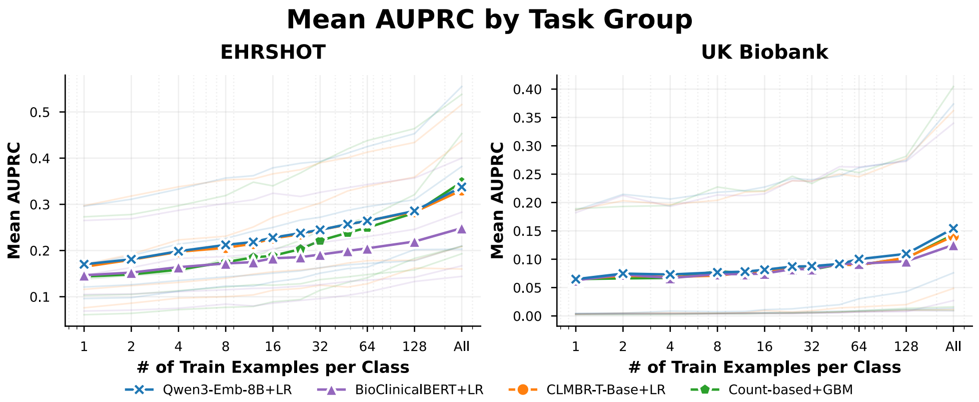

Figure S6 Few-Shot Performance on EHRSHOT and UKB. Macro-averaged area under the precision-recall curve (AUPRC) performance across all subtasks of EHRSHOT (left) and UK Biobank (right). Blurred lines show averaged AUPRC values for the different task groups.

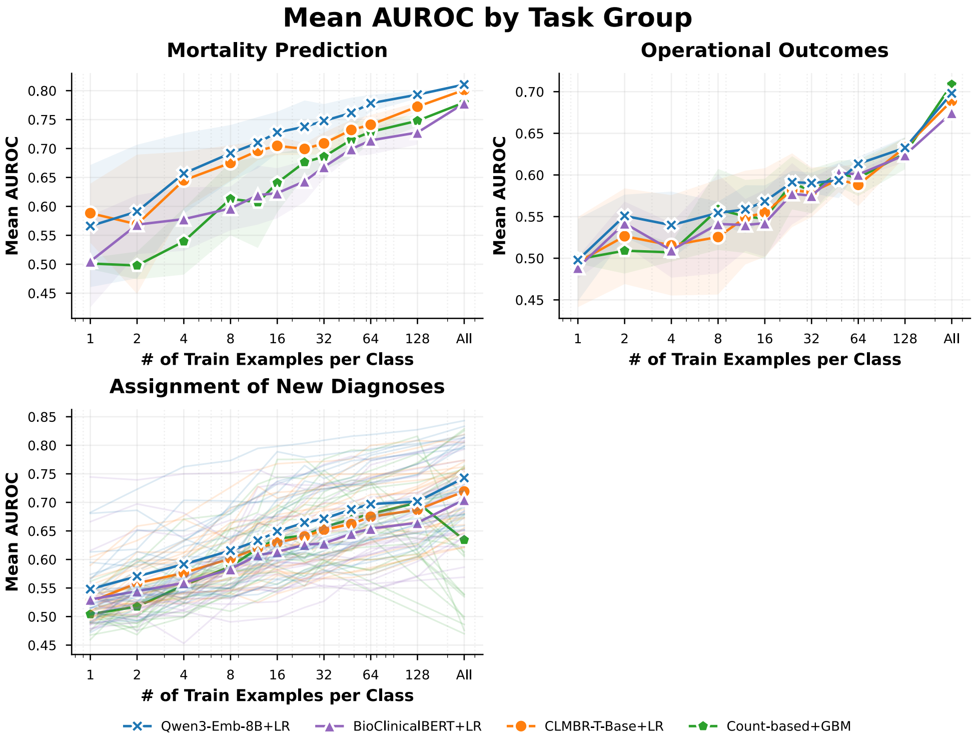

Figure S7 Few-Shot AUROC Performance on UKB. Mean area under the receiver operating characteristic curve (AUROC) performance across subtasks for three task groups (bold). Blurred lines show averages across five bootstrapped runs using different seeds. Shaded regions show standard deviation.

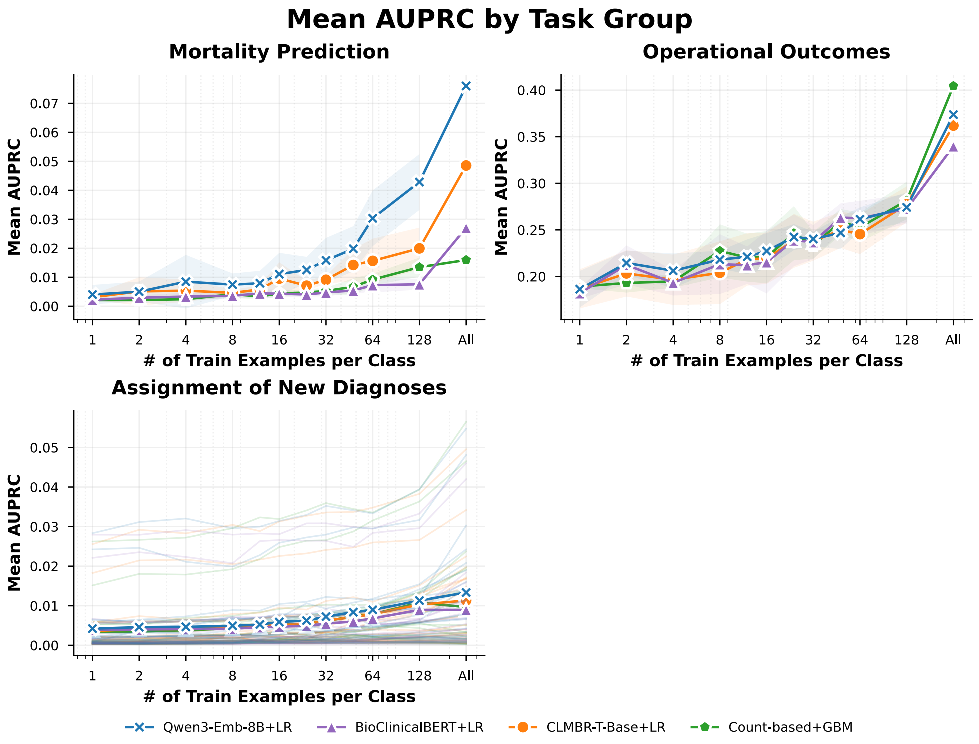

Figure S8 Few-Shot AUPRC Performance on UKB. Mean area under the precision-recall curve (AUPRC) performance across subtasks for three task groups (bold). Blurred lines show averages across five bootstrapped runs using different seeds. Shaded regions show standard deviation.

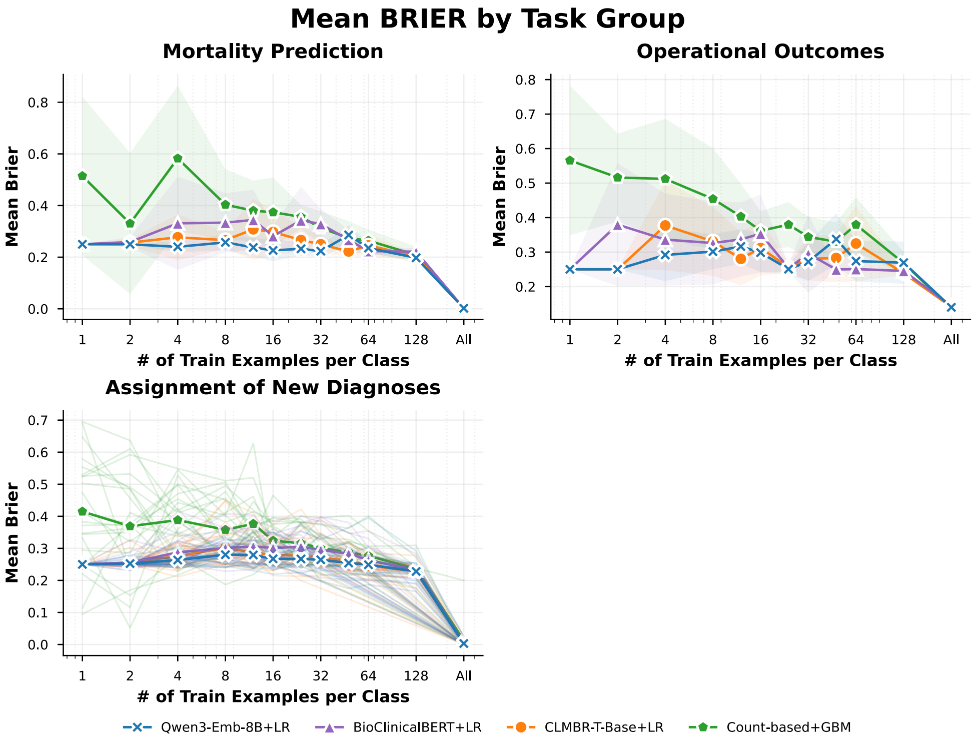

Figure S9 Few-Shot Brier Score on UKB. Mean Brier score across subtasks for three task groups (bold). Blurred lines show averages across five bootstrapped runs using different seeds [45]. Shaded regions show standard deviation.

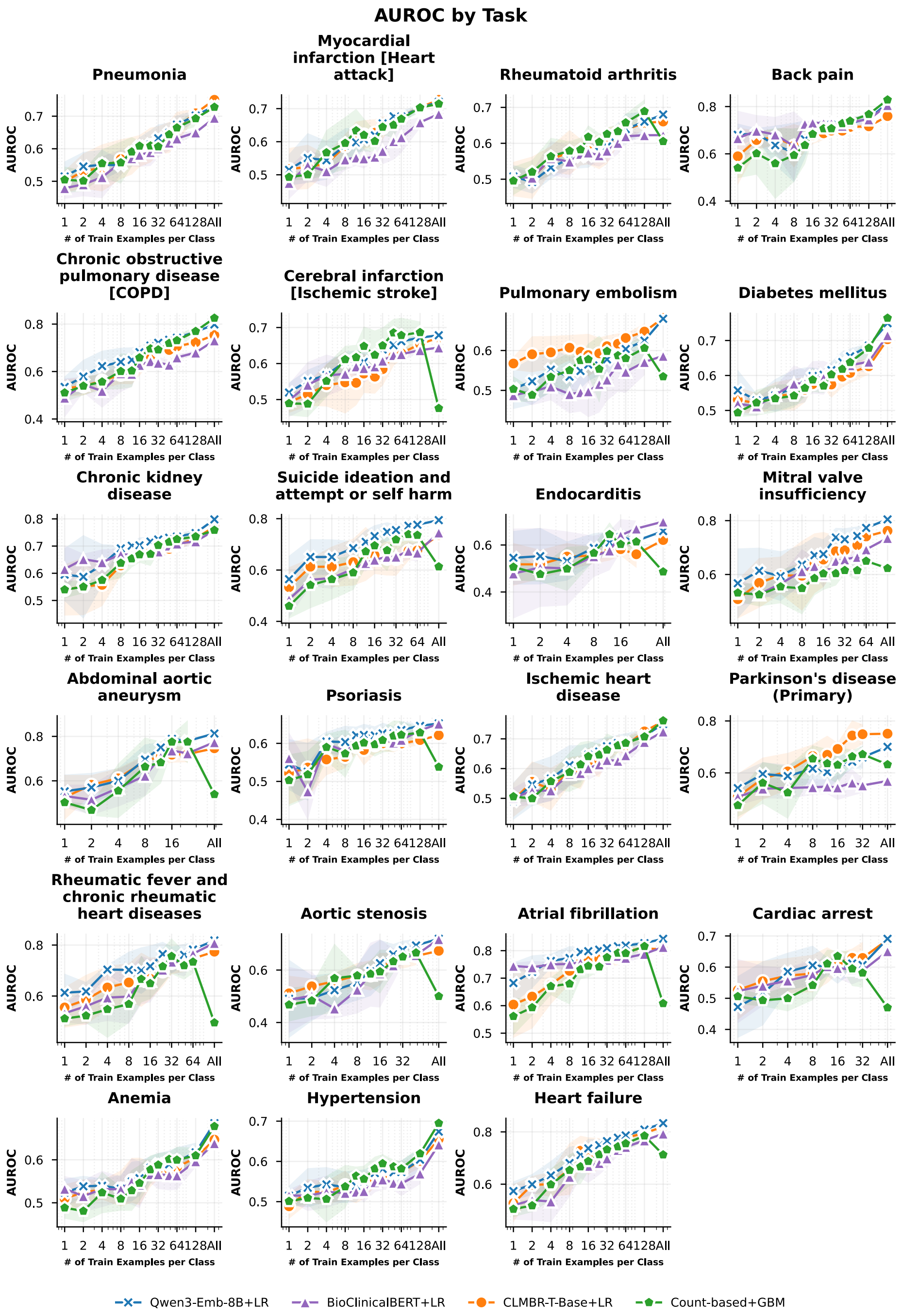

Figure S10 Disease Onset AUROC Performance on UKB. Area under the receiver operating characteristic curve (AUROC) performance with standard deviation across five few-shot replicates for all assignment-of-new-diagnosis tasks.

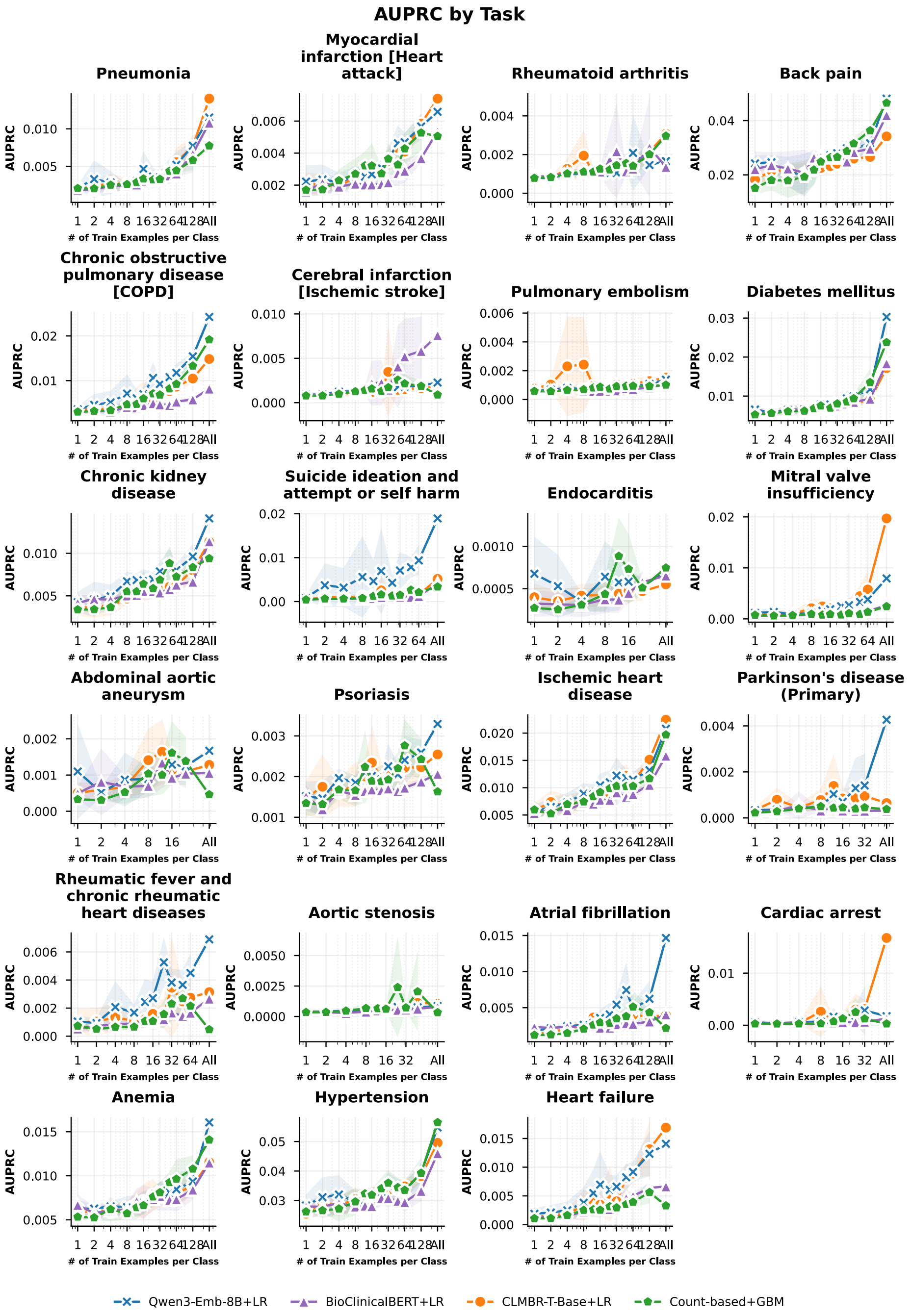

Figure S11 Disease Onset AUPRC Performance on UKB. Area under the precision-recall curve (AUPRC) performance with standard deviation across five few-shot replicates for all assignment-of-new-diagnosis tasks.

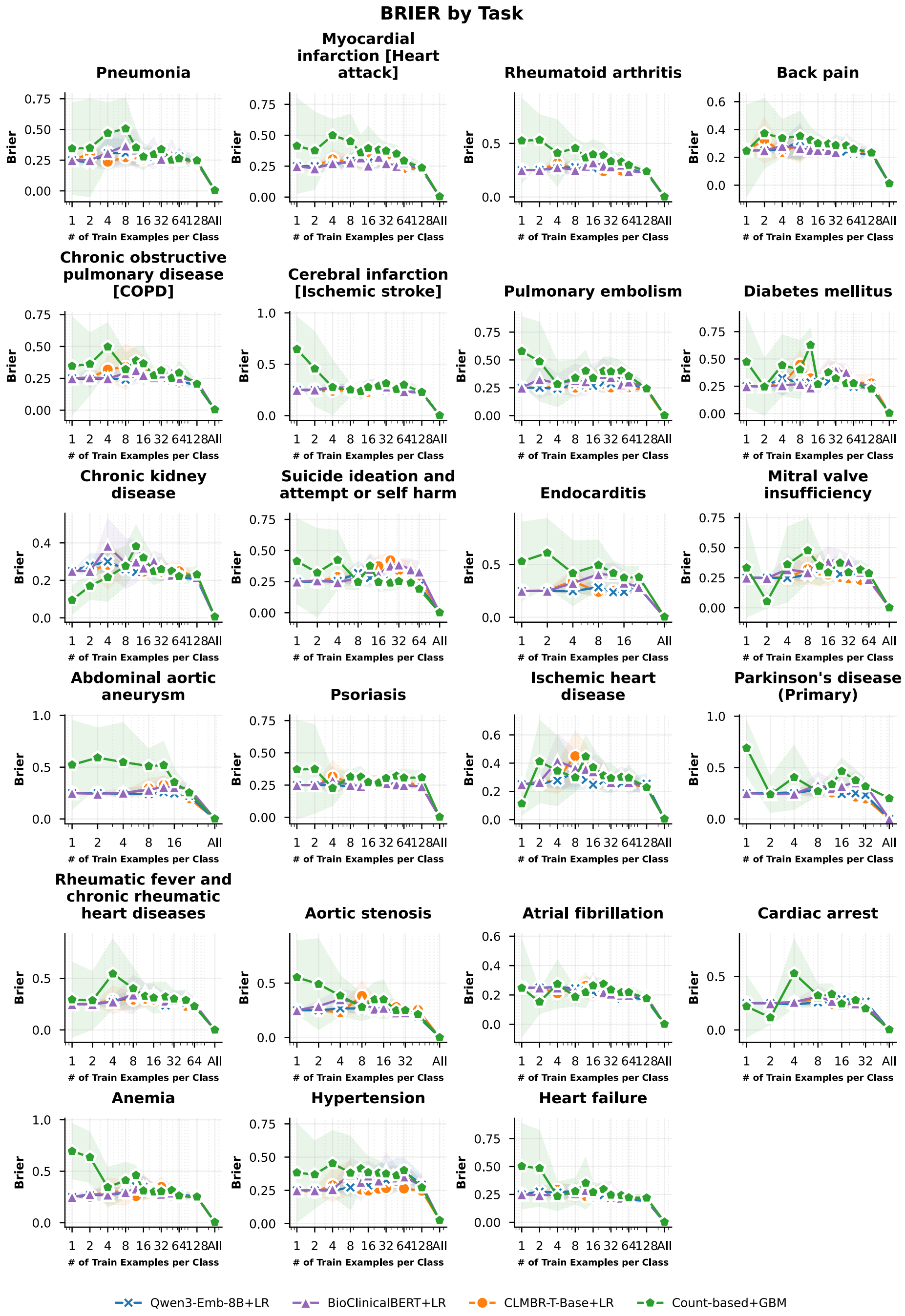

Figure S12 Disease Onset Brier Score on UKB. Brier score with standard deviation across five few-shot replicates for all assignment-of-new-diagnosis tasks.

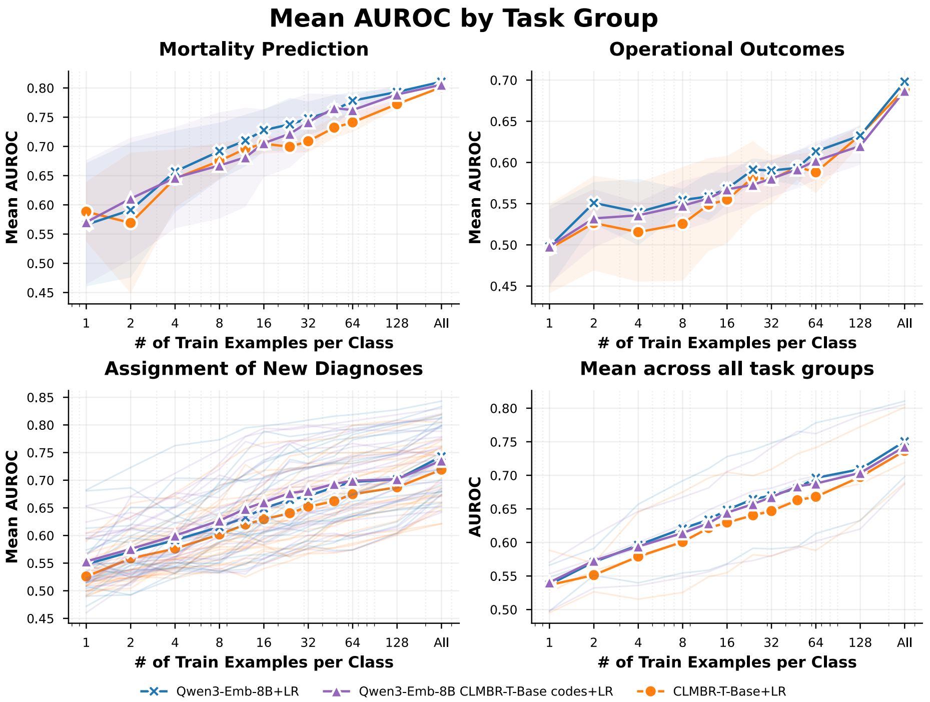

Figure S13 Few-Shot AUROC Performance for Sensitivity Analysis on UKB. Mean area under the receiver operating characteristic curve (AUROC) performance across subtasks for three task groups (bold) and macro-averaged AUROC performance across all subtasks on the UK Biobank. Results are reported for Qwen3-Emb-8B using all UKB codes, Qwen3-Emb-8B restricted to medical codes mappable to the EHR foundation model CLMBR-T-Base, and the CLMBR-T-Base model. Shaded regions indicate standard deviation. Blurred lines for assignment of new diagnoses represent averages across five bootstrapped runs using different seeds. The blurred lines for the mean across all task groups represent the averaged AUROC values of the different task groups.

1.5 Additional Performance Results for Encoder and Decoder Models

Table S18 Performance for All Examples on EHRSHOT. Macro-averaged AUROC performance and bootstrapped 95% confidence intervals for the frozen Qwen3-Emb-8B encoder baseline and the LoRA-tuned Qwen encoder and decoder variants at $k = 128$. Fine-tuning via LoRA did not improve over the frozen Qwen3-Emb-8B baseline. Among the tuned variants, the fine-tuned decoder slightly outperformed the fine-tuned encoder, but both remained below the frozen baseline and the decoder required substantially higher computational cost.

**Model**	**Operational Outcomes**	**Anticipating Lab Test Results**	**Assignment of New Diagnoses**	**Anticipating Chest X-ray Findings**	**Macro Avg. Across Task Groups**
**Encoder models**					
Qwen3-Emb-8B	0.767 $_{.726\text{-}.803}$	0.770 $_{.749\text{-}.789}$	0.702 $_{.614\text{-}.783}$	0.695 $_{.654\text{-}.733}$	0.733 $_{.686\text{-}.777}$
Qwen3-Emb-8B (LoRA)	0.736 $_{.693\text{-}.776}$	0.819 $_{.804\text{-}.834}$	0.667 $_{.573\text{-}.751}$	0.646 $_{.605\text{-}.686}$	0.717 $_{.669\text{-}.762}$
**Decoder models**					
Qwen3-8B (LoRA)	0.692 $_{.651\text{-}.732}$	0.848 $_{.835\text{-}.860}$	0.711 $_{.620\text{-}.801}$	0.626 $_{.588\text{-}.664}$	0.719 $_{.673\text{-}.764}$

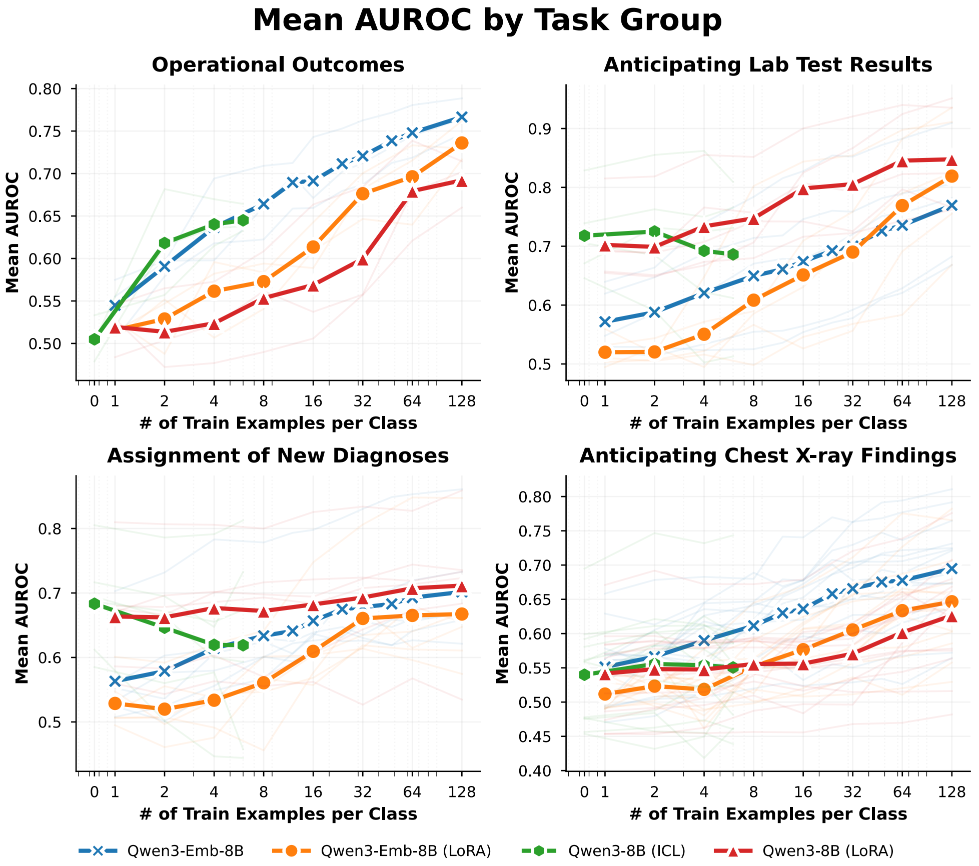

Figure S14 Few-Shot AUROC Performance of Encoder and Decoder Models on EHRSHOT by Task Group. Mean area under the receiver operating characteristic curve (AUROC) across subtasks for the four EHRSHOT task groups. Blurred lines show averages across five bootstrapped runs using different seeds. The decoder ICL curves are shown for 0, 2, 4, and 6 shots only because larger ICL settings were not computationally feasible.

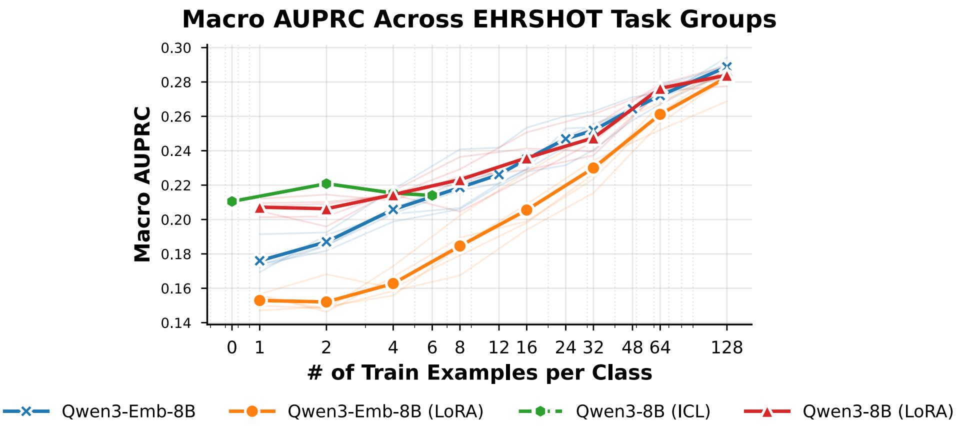

Figure S15 Few-Shot AUPRC Performance of Encoder and Decoder Models on EHRSHOT. Macro-averaged area under the precision-recall curve (AUPRC) across all EHRSHOT subtasks for zero to 128 training examples per class, comparing the frozen encoder baseline, the LoRA-tuned encoder, decoder ICL at 0, 2, 4, and 6 shots, and the LoRA-tuned decoder.

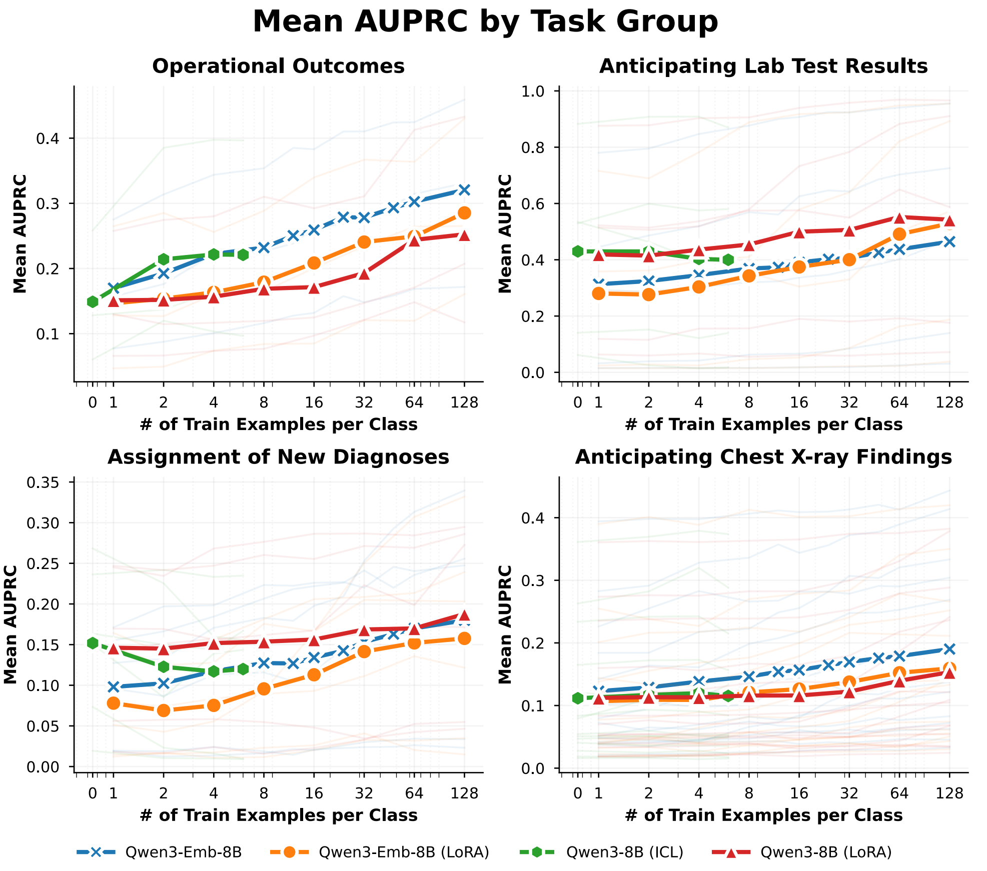

Figure S16 Few-Shot AUPRC Performance of Encoder and Decoder Models on EHRSHOT by Task Group. Mean area under the precision-recall curve (AUPRC) across subtasks for the four EHRSHOT task groups. Blurred lines show averages across five bootstrapped runs using different seeds. Decoder ICL beyond 6 shots was not computationally feasible and is therefore not shown.
